# A metagenomic investigation of phytoplasma diversity in Australian vegetable growing regions

**DOI:** 10.1099/mgen.0.001213

**Published:** 2024-03-06

**Authors:** Bianca Rodrigues Jardim, Cherie Gambley, Lucy T. T. Tran-Nguyen, Craig Webster, Monica Kehoe, Wycliff M. Kinoti, Samantha Bond, Richard Davis, Lynne Jones, Nandita Pathania, Murray Sharman, Toni Chapman, Brendan C. Rodoni, Fiona E. Constable

**Affiliations:** 1 School of Applied Systems Biology, La Trobe University, Bundoora, Victoria, Australia; 2 Agriculture Victoria Research, Department of Energy, Environment and Climate Action, AgriBio, Bundoora, Victoria, Australia; 3 Horticulture and Forestry Science, Department of Agriculture and Fisheries Maroochy Research Facility, Nambour, Queensland, Australia; 4 Plant Health Australia, Deakin, Australian Capital Territory, Australia; 5 Diagnostic Laboratory Services, Department of Primary Industries and Regional Development, South Perth, Western Australia, Australia; 6 Biosecurity and Animal Welfare, Department of Industry, Tourism and Trade, Darwin, Northern Territory, Australia; 7 Northern Australia Quarantine Strategy, Department of Agriculture, Fisheries and Forestry, Canberra, Australian Capital Territory, 2601, Australia; 8 Department of Agriculture and Fisheries, Mareeba, Queensland, Australia; 9 Department of Agriculture and Fisheries, Ecosciences Precinct, Dutton Park, Queensland 4102, Australia; 10 Biosecurity and Food Safety, New South Wales Department of Primary Industries, Elizabeth Macarthur Agricultural Institute (EMAI), Menangle, New South Wales, 2567, Australia

**Keywords:** ANI, *Candidatus* Phytoplasma australasiaticum, *Candidatus* Phytoplasma stylosanthis, draft genomes, metagenomic assembled genomes (MAGs), unclassified phytoplasmas

## Abstract

In this study, metagenomic sequence data was used to investigate the phytoplasma taxonomic diversity in vegetable-growing regions across Australia. Metagenomic sequencing was performed on 195 phytoplasma-positive samples, originating either from historic collections (*n*=46) or during collection efforts between January 2015 and June 2022 (*n*=149). The sampled hosts were classified as crop (*n*=155), weed (*n*=24), ornamental (*n*=7), native plant (*n*=6), and insect (*n*=3) species. Most samples came from Queensland (*n*=78), followed by Western Australia (*n*=46), the Northern Territory (*n*=32), New South Wales (*n*=17), and Victoria (*n*=10). Of the 195 draft phytoplasma genomes, 178 met our genome criteria for comparison using an average nucleotide identity approach. Ten distinct phytoplasma species were identified and could be classified within the 16SrII, 16SrXII (PCR only), 16SrXXV, and 16SrXXXVIII phytoplasma groups, which have all previously been recorded in Australia. The most commonly detected phytoplasma taxa in this study were species and subspecies classified within the 16SrII group (*n*=153), followed by strains within the 16SrXXXVIII group (‘*Ca*. Phytoplasma stylosanthis’; *n*=6). Several geographic- and host-range expansions were reported, as well as mixed phytoplasma infections of 16SrII taxa and ‘*Ca*. Phytoplasma stylosanthis’. Additionally, six previously unrecorded 16SrII taxa were identified, including five putative subspecies of ‘*Ca*. Phytoplasma australasiaticum’ and a new putative 16SrII species. PCR and sequencing of the 16S rRNA gene was a suitable triage tool for preliminary phytoplasma detection. Metagenomic sequencing, however, allowed for higher-resolution identification of the phytoplasmas, including mixed infections, than was afforded by only direct Sanger sequencing of the 16S rRNA gene. Since the metagenomic approach theoretically obtains sequences of all organisms in a sample, this approach was useful to confirm the host family, genus, and/or species. In addition to improving our understanding of the phytoplasma species that affect crop production in Australia, the study also significantly expands the genomic sequence data available in public sequence repositories to contribute to phytoplasma molecular epidemiology studies, revision of taxonomy, and improved diagnostics.

## Data Summary

The phytoplasma genomes analysed in this study were downloaded from the NCBI or sequenced for this study. The list of genomes that were already available on the NCBI are listed in Tables S1 and S2, available in the online version of this article, while those added from this publication are listed in Table S1 only and were submitted under the BioProject PRJNA980440. Tables S1 and S2; Fig. S1 are available with the online version of this article.

**Impact Statement**

Phytoplasmas are unculturable, plant pathogenic bacteria that infect and impact yield of many agriculturally important plant species. In this study, 16S rRNA gene detection and sequencing for triaging was coupled with metagenomic sequencing to determine the diversity of phytoplasma taxa and associated diseases in Australian vegetable growing regions. It is the first study to use metagenomic analysis to improve the understanding of phytoplasma diversity in Australia. None of the phytoplasma taxa that were detected were exotic to Australia, but host- and geographic-range expansions were recorded for some. Since the metagenomic approach obtains DNA sequences from all organisms in a sample, the identification of plant and insect host families, genera, or species was possible by DNA barcode analysis when they were undetermined based on morphology. This study has analysed the largest number of phytoplasma whole genomes to date (*n*=195) and significantly contributes to the available sequence data for these bacteria. The sequence and metadata provided in this study offer an improved understanding of the phytoplasma taxa present in the different states and territories of Australia, and contributes to improving phytoplasma taxonomy, molecular epidemiology, and diagnostics.

## Introduction

Phytoplasmas are a diverse monophyletic clade of unculturable bacteria in the class *Mollicutes* within the provisional genus ‘*Candidatus* Phytoplasma’ [1]. They are phloem-limited plant pathogenic bacteria that are transmitted by phloem-feeding hemipteran insects [2]. Diseases associated with phytoplasma infections have been described in over 700 plant hosts globally from agriculturally and horticulturally important crops, ornamental plants, weeds, and native plants [3,5].

In Australia, phytoplasma-like symptoms were first recorded in the 1900s from tomato (Tomato Big Bud, TBB) [6], lucerne (Lucerne Witches’-Broom) [7], and pasture legumes (Legume Little Leaf) [8]. All were thought to be vector-transmitted viruses until mycoplasma-like structures were identified by electron microscopy in fabaceous plants showing symptoms of little leaves and spindled stems [9]. By the 1990s, molecular detections of phytoplasmas were being made globally [10], including in Australia [11], by PCR amplification of the 16S rRNA gene region. The molecular, PCR-based techniques facilitated the screening of many more plants for phytoplasma infections than was previously possible by microscopy and serological techniques. These PCR-based techniques involved restriction fragment length polymorphism (RFLP) analysis and/or sequencing for phytoplasma characterization and identification. Phytoplasma surveys were done in Australia using these molecular-based approaches and a large diversity of phytoplasma taxa affecting grains, legumes, fruit and vegetable crops, ornamentals, native plants, weeds and putative phytoplasma vectors were uncovered [12,18]. To date, twelve phytoplasma 16 Sr groups and five ‘*Candidatus* Phytoplasma’ representatives have been described from Australia [19,25]. Members within the 16SrII group are the most commonly detected phytoplasma in Australia, affecting a large number of host species [12,13, 16, 26]. However, members within the 16SrXII group have been described as the most economically important based on their association with yield-reducing diseases in high-value crops such as grapevines and strawberries [26,28]. Competent vector species remain to be confirmed for many of the phytoplasma taxa present in Australia, although *Orosius argentatus* has been shown to transmit diseases associated with 16SrII phytoplasmas [8,9, 29, 30].

Phytoplasma diversity and taxonomic analyses, including studies in Australia, have largely relied on the 16S rRNA gene sequence, which only offers low-resolution analyses of inter- and intraspecies diversity [1,12, 31, 32]. Diversity assessments and species delimitation studies have also involved higher-resolution analyses of three to nine additional housekeeping genes for multilocus sequence typing (MLST) and analysis (MLSA) [24,33, 34]. With the decreasing cost of high-throughput sequencing (HTS) and increasing sequence data outputs, as well as the advancements in bioinformatic tools for metagenomic data assessments, phytoplasma genomes have increasingly been used to understand their taxonomy [31] and biology [35]. Obtaining draft or complete phytoplasma genomes allows for higher-resolution analyses than those based on one or a few genes for diversity analyses [36]. Additionally, by applying genome-based species delimitation thresholds and criteria specified for culturable bacteria, taxonomic boundaries between phytoplasma strains can be identified [36,37].

The aim of this study was to assess and update the species and genetic diversity of phytoplasmas in vegetable growing regions in the various states and territories of Australia using whole-genome-based approaches. To this end, (i) plants displaying phytoplasma associated symptoms and insects were collected from vegetable growing regions in Australia and screened by PCR for the presence of phytoplasma infections, (ii) DNA from key historical phytoplasma strains from previous phytoplasma surveys in Australia were obtained, and (iii) a metagenomic approach was used to obtain draft phytoplasma genome assemblies to be used for genome-based investigations into the phytoplasma taxa infecting the samples, and to identify the host when the plant or insect host identity was inconclusive based on morphology. The results of this study demonstrate the applications, benefits, and challenges of applying metagenomic sequencing to phytoplasma diversity analyses.

## Methods

### Sample collection, total nucleic acid extraction, plant tissue preservation, and DNA quantification

Plant samples with phytoplasma-associated symptoms, including little leaf, yellowing, phyllody, and/or stunting that were collected Australia-wide between March 2019 and June 2022 were sent to the laboratory in Melbourne, Victoria (VIC) for analysis (Table S1). Total nucleic acid was extracted from samples using an iodixanol-based phytoplasma enrichment procedure [38] or a modified CTAB-DNeasy protocol without RNase treatment [39] (Table S1 and S2). Petioles, whole leaves or leaf veins were used in total nucleic acid extractions for most samples. Phloem scrapings were sampled for woody material (e.g. *Melaleuca* spp. and *Vitaceae* spp.). When possible, a subsample of the plant material was freeze dried for at least 72 h at −50 °C in individually labelled screw cap tubes using the FreeZone 2.5 Liter Benchtop Freeze Dry System (Labconco, MO, USA) and deposited in the Victorian plant pathology herbarium (VPRI) (Table S1). Insect samples were supplied as DNA extracts (Table S1) and had been collected by suction trapping and sweep netting in Jennings, New South Wales (NSW) and Palmerston, Northern Territory (NT), respectively. Insect collections were done in these areas as plants displaying phytoplasma-associated symptoms were present nearby. Phytoplasma-positive samples collected prior to 2019 that are held at The Northern Territory Department of Industry, Tourism and Trade (NT DITT), Darwin, NT, Australia and Department of Agriculture and Fisheries, Mareeba, Queensland (QLD), Australia were also supplied as total nucleic acid extracts (Table S1).

DNA quantity was estimated using a Qubit 2.0 fluorometer (Thermo Fisher Scientific, MA, USA) with the Qubit 1X dsDNA HS Assay Kit (Thermo Fisher Scientific, MA, USA). All DNA samples were stored at −20 °C.

### Phytoplasma screening and preliminary identification by universal phytoplasma 16Sr PCR, and Sanger sequencing

Screening for PCR inhibitors was done using PCR primers for the generic amplification of the bacterial 16S rRNA gene [40]. A nested PCR assay using P1/P7 and R16F2n/m23sr primer pairs were used to screen recent samples for phytoplasma infection and to confirm phytoplasma presence in total nucleic acid extracts from samples collected prior to 2019 [41]. These primers all bind to the phytoplasma 16S rRNA gene, apart from the P7 primer, which binds to the 5′ region of the 23S rRNA gene. The R16F2n/m23sr amplicon of some samples were cloned and screened according to [24] when a poor Sanger sequencing quality was observed in the forward and/or reverse read (Table S1) [24]. All PCR amplicons were visualized by electrophoresis through 1 % agarose gels stained with SYBR Safe DNA gel stain (Thermo Fisher Scientific, MA, USA). PCR amplicons of the expected size were purified and directly Sanger sequenced (Macrogen, Seoul, South Korea). The identities of the Sanger sequenced PCR amplicons were determined by blastn analysis [42] at the NCBI (https://www.ncbi.nlm.nih.gov/, last accessed 30 July 2022). During the blastn analyses, the top hit with the 16S rRNA gene of a ‘*Ca.* Phytoplasma’ species reference strain was used to determine the identity of the sample investigated based on the top bit score, percent identity, and e-value, as well as considering the query coverage.

### Library preparation and sequencing

Libraries were prepared with fragment sizes between 300 and 500 bp by following the manufacturer’s protocols of the NEXTFLEX Rapid XP DNA-Seq Kit (PerkinElmer, MA, USA) with the Unique Dual Index (UDI) barcodes (PerkinElmer, MA, USA) or the Nextera DNA Flex Library Preparation Kit (Illumina, CA, USA) with the IDT for Illumina Nextera DNA Unique Dual (UD) Indexes (Illumina, CA, USA) (Table S1). The libraries were pooled and size-selected prior to sequencing according to [38] and sequenced with Illumina platforms including the MiSeq (2×250 bp), HiSeq2000 (2×150 bp), NovaSeq 6000 on an SP flow cell (2×250 bp), or NovaSeq 6000 on an S1 flow cell (2×150 bp). All the library pools sequenced on the NovaSeq 6000 platform were treated with the Illumina Free Adapter Blocking Reagent (Illumina, CA, USA) according to the manufacturer protocols prior to HTS to mitigate aberrant sequencing results caused by the presence of free adapters.

The phytoplasma genome sequences of 25 phytoplasma-positive samples have been used in previous studies [24,37, 38]. The genome sequences and metadata associated with these samples were included in this study, however, they were gathered during the sample collection period of this study (Table S2).

### Sequence data analyses

#### Read quality filtering, metagenomic assembly, and identification of phytoplasma-derived contigs and gene annotations

Illumina read filtering and adapter trimming for each sample was done using FastP [43], removing reads shorter than 50 bp and with a Phred quality score (Q score) below Q20. The trimmed reads were used in a metagenomic assembly pipeline according to [38], which implements metaSPAdes version 3.15.2 [44,45]. Phytoplasma-derived contigs were identified and retrieved using blast+v2.11.0 [42] and a custom *grep script, respectively. Contigs shorter than 500 bp were removed using the reformat.sh* script implemented in the BBMap v.38.61b software suite [46]. The phytoplasma genomes were analysed in metaQUAST [47] to estimate the genome N50 values. Protein coding, tRNA, and rRNA genes were annotated and counted using Prokka [48], specifying RNAmmer for 5S, 16S, and 23S rRNA gene annotations [49].

#### Selection of phytoplasma genomes for comparative genomic assessments

All phytoplasma genomes available on the NCBI (https://www.ncbi.nlm.nih.gov/) were downloaded, apart from the ‘*Ca*. Phytoplasma citri’ (synonym ‘*Ca*. Phytoplasma aurantifolia’) genome under the RefSeq accession no. GCF_002009625.1 (Table S2). Instead, the re-sequenced assembly of this strain described in [37] was used (Table S2).

The phytoplasma genomes obtained in this study were analysed differently based on the total genome size recovered to mitigate spurious results related to highly incomplete and fragmented genomes. The number of tRNA gene sequences annotated were used to estimate the completeness of the phytoplasma assemblies and, therefore, their suitability for the downstream genome-based analyses. Phytoplasma genomes that were larger than 300,000 bp and which encoded 13 or more tRNA genes were used in further whole-genome-based assessments to classify the phytoplasma strains (Table S1). For genomes smaller than 300,000 bp and/or encoding fewer than 13 tRNA genes, only the 16S rRNA gene was used to classify the phytoplasma strain within a 16 Sr group (Table S1).

#### Whole-genome analyses

Whole-genome comparisons were performed for all the phytoplasma genomes by average nucleotide identity (ANI) analysis using the ANI with MUMmer (ANIm) algorithm in pyani version 0.2.10 [50]. The pyani heatmap output was manually overlaid with blastn results to characterize clusters, which did not contain a representative genome.

For further investigation into phytoplasma samples that did not clearly cluster with reference sequences in the initial ANI analysis, the coverage of aligned genomic segments in each pairwise comparison was analysed along with publicly available phytoplasma reference genomes using pyani version 0.20.10. These values are referred to as the alignment fraction (AF).

In cases where a historic strain was previously classified using *in vitro RFLP but appeared to be a different species based on the maximum-likelihood tree,* the recognition sites for 14 of the 17 restriction enzymes used in phytoplasma subgroup classification were visualized in Geneious Prime version 2022.2.2 (https://www.geneious.com/prime/). The three restriction enzymes that were not used as they are unavailable on Geneious Prime version 2022.2.2 are: *Bfa*I, *Bst*UI (*Tha*I), and *Ssp*I.

#### Identification of mixed phytoplasma infections using whole-genome analyses

Samples which demonstrated high ANI values (>90 % ANI) with more than one representative phytoplasma genome were considered to have a mixed phytoplasma infection of the phytoplasma taxa for which genome sequence data was available. The 16S rRNA gene sequence analyses, including those of the cloned PCR amplicons, as well as the number of annotated tRNA genes were revisited for the mixed infection samples identified during the blastn analyses.

#### Identification of unknown host species

The host family, genus, or species of some plant hosts and two insect hosts were unconfirmed based on external morphology prior to total nucleic acid extraction (Table S1). To confirm the identities of the insects to the species-level, contigs encoding *cytochrome c oxidase subunit 1* (*coI*) were analysed [51]. To confirm the family, genus, and/or species of the unknown plant hosts, the contigs encoding *maturase K* (*matK*) and *ribulose-bisphosphate carboxylase* (*rbcL*) genes were analysed [52,53]. These barcoding genes were obtained from the metagenome assemblies using a custom grep script to identify the genes and their identities from the blastn results. A publicly available accession was listed as the top blastn hit when it produced the highest bit-score, lowest e-value, and highest percentage identity. In cases where multiple species in the same genus were considered the top blastn hit, only the genus was recorded for the sample and the species names were considered undetermined. In cases where multiple genera were listed as top blastn hits, the family of these samples were recorded, and the genus and species names were considered undetermined. The detection location of host species listed as top hits were determined based on searches performed at the Australasian Virtual Herbarium website (https://avh.chah.org.au/, last accessed March 2023), with hosts that are not known to be present in Australia removed from the list.

## Results and discussion

### Phytoplasma-positive sample information

A total of 195 samples were collected between 1998 and 2022 and were either confirmed or suspected to be infected with phytoplasma based on previous PCR-based analyses (sequence similarity and RFLP) or disease symptoms. These samples were subsequently confirmed to be positive for phytoplasma by PCR and direct Sanger sequencing of the amplicon generated using the universal phytoplasma 16S rRNA primers. Most of the samples were collected from QLD (*n*=78), followed by Western Australia (WA, *n*=46), NT (*n*=32), NSW (*n*=29), and VIC (*n*=10) (Tables 1 and S1).

**Table 1.** Summary of all host species, genera, and families investigated in this study and associated metadata organized according to their sampling location in Australia (state/territory and closest town/city). Metadata recorded included the sample names, sampling years, and the phytoplasma taxa identified based on blastn or ANI analyses performed in this study. Original sample names, if provided, are recorded alongside the corresponding ‘BAWM’ name in Table S1

Location	Host family	Host species/genus	Phytoplasma taxa identified by ANI or 16S rRNA blastn	Sampling year(s)	Sample name(s)
**NSW**					
**Jennings**					
	Cicadellidae	*Orosius argentatus*	'*Ca*. P. stylosanthis''*Ca*. P. australasiaticum subsp. australasiaticum'	20212021	BAWM-342B*BAWM-343A*
	Solanaceae	*Capsicum annuum*	'*Ca*. P. australasiaticum subsp. australasiaticum''*Ca*. P. australasiaticum subsp. ipomoeae'	2020202020202020202020202021202120212021201720202021	BAWM-117BAWM-118BAWM-119BAWM-124 BAWM-126BAWM-131aBAWM-192BAWM-194 BAWM-195BAWM-203BAWM-054BAWM-135BAWM-193a-F1
	Solanaceae	*Solanum lycopersicum*	'*Ca*. P. australasiaticum subsp. australasiaticum'	2017201720202020202020202020202020202020202020212021	BAWM-049BAWM-053BAWM-113BAWM-114BAWM-115BAWM-116BAWM-125aBAWM-127BAWM-128BAWM-134aBAWM-134bBAWM-204BAWM-205
**Mudgee**					
	Vitaceae	*Vitis viniferacv*. Riesling	'*Ca*. P. australasiaticum subsp. australasiaticum'	2021	BAWM-233
**Wallicia**					
	Solanaceae	*Capsicum annuum*	'*Ca*. P. australasiaticum subsp. australasiaticum'	2020	BAWM-131a
**nt**					
**Berry Springs**					
	Fabaceae	*Vigna unguiculata* ssp. *sesquipedalis*	'*Ca*. P. australasiaticum subsp. ipomoeae'	20192019	BAWM-082BAWM-083
**Darwin**					
	Asteraceae	*Gynura crepioides* (Okinawa Spinach)	'*Ca*. P. australasiaticum' taxon 1	2021	BAWM-338
	Convolvulaceae	*Ipomoea* sp.	New 16SrII species	2021	BAWM-339
	Solanaceae	*Solanum melongena*	'*Ca*. P. australasiaticum' taxon 1'*Ca*. P. australasiaticum subsp. australasiaticum'	20042005200520052005	BAWM-293BAWM-295BAWM-296BAWM-298BAWM-297
**Girraween**					
	Solanaceae	*Solanum melongena*	'*Ca*. P. australasiaticum' taxon 1	200420042004	BAWM-289BAWM-290BAWM-291
**Howard Island**					
	Convolvulaceae	*Ipomoea batatas*	'*Ca*. P. australasiaticum subsp. australasiaticum'	1998	BAWM-259
**Humpty Doo**					
	Cucurbitaceae	*Luffa acutangula*	'*Ca*. P. australasiaticum subsp. ipomoeae'	20182018	BAWM-300BAWM-301
	Cucurbitaceae	*Momordica charantia*	'*Ca*. P. australasiaticum subsp. ipomoeae'	2018	BAWM-303
	Cucurbitaceae	*Trichosanthes cucumerina*	'*Ca*. P. australasiaticum' taxon 3	2018	BAWM-306
	Fabaceae	*Vigna unguiculata* ssp. *sesquipedalis*	'*Ca*. P. fabacearum'	2004	BAWM-286
**Katherine**					
	Caricaceae	*Carica papaya*	'*Ca*. P. stylosanthis'	1998	BAWM-249
	Convolvulaceae	*Bonamia pannosa*	'*Ca*. P. bonamiae''*Ca*. P. bonamiae'	19981998	BAWM-225BAWM-226
	Malvaceae	*Waltheria* sp.	*Waltheria* Little Leaf (WaLL) phytoplasma	1998	BAWM-227
**Lambells lagoon**					
	Cucurbitaceae	*Luffa acutangula*	*'Ca*. P. australasiaticum subsp. ipomoeae'	2019	BAWM-081
**Marrakai**					
	Cucurbitaceae	*Luffa acutangula*	*'Ca*. P. australasiaticum subsp. ipomoeae'	2016	BAWM-299
	Cucurbitaceae	*Momordica charantia*	*Vigna* Little Leaf (ViLL) phytoplasma	20212021	BAWM-336BAWM-337
**Palmerston**					
	Cicadellidae	*Orosius orientalis*	'*Ca*. P. australasiaticum subsp. ipomoeae'	2020	BAWM-232
	Fabaceae	*Arachis hypogaea*	*‘Ca*. P. australasiaticum subsp. ipomoeae'	2019	BAWM-080
	Fabaceae	*Crotalaria* sp.	*'Ca*. P. australasiaticum subsp. ipomoeae'	2020	BAWM-164
	Solanaceae	*Solanum* sp.	Mixed infection ('*Ca*. P. stylosanthis and'*Ca*. P. australasiaticum' taxon 1)	2019	BAWM-079*
**Unknown**					
	Solanaceae	*Solanum melongena*	'*Ca*. P. australasiaticum subsp. australasiaticum'	2004	BAWM-287
	Solanaceae	*Solanum* sp.	'*Ca*. P. australasiaticum' taxon 1	2018	BAWM-302*
**QLD**					
**Atherton**					
	Fabaceae	*Stylosanthes scabra*	'*Ca*. P. stylosanthis'Mixed infection ('*Ca*. P. stylosanthis and '*Ca*. P. australasiaticum subsp. ipomoeae’)	19981998199819981998	BAWM-254BAWM-256BAWM-258BAWM-253BAWM-257
**Bowen**					
	Solanaceae	*Capsicum annuum*	'*Ca*. P. fabacearum'	2020	BAWM-168
**Brisbane**					
	Solanaceae	*Solanum lycopersicum*	'*Ca*. P. australasiaticum subsp. australasiaticum'	20212021	BAWM-217BAWM-218
**Bundaberg**					
	Malvaceae	*Sida* sp.	'*Ca*. P. australasiaticum subsp. australasiaticum'	2019	BAWM-037*
**Cairns**					
	Lecythidaceae	*Planchonia careya*	'*Ca*. P. planchoniae'	2020	BAWM-156b
	Myrtaceae	*Melaleuca* sp.	'*Ca*. P. melaleucae'	2020	BAWM-155a
**Croydon**					
	Fabaceae	*Glycine max*	'*Ca*. P. fabacearum'	2017	BAWM-350
**Dalby**					
	Fabaceae	*Vigna radiata*	*'Ca*. P. australasiaticum subsp. ipomoeae'	2017	BAWM-349
**Davies Creek**					
	Pedaliaceae	*Phyllanthus fuernrohrii*	*'Ca*. P. australasiaticum subsp. ipomoeae'	2018	BAWM-313
**Dimbulah**					
	Solanaceae	*Solanum lycopersicum*	*'Ca*. P. australasiaticum subsp. australasiaticum''*Ca*. P. australasiaticum subsp. ipomoeae'	200220022002200220022002200220022002	BAWM-260BAWM-261BAWM-264BAWM-265BAWM-266BAWM-269BAWM-262BAWM-263BAWM-267
**Emerald**					
	Fabaceae	*Cajanus cajan*	*'Ca*. P. australasiaticum subsp. ipomoeae'	2020	BAWM-351
**Gatton**					
	Solanaceae	*Capsicum annuum*	*'Ca*. P. australasiaticum subsp. ipomoeae'	20022002	BAWM-276BAWM-277
**Glen Aplin**					
	Solanaceae	*Capsicum annuum*	'*Ca*. P. australasiaticum subsp. australasiaticum''*Ca*. P. fabacearum'	202120212021	BAWM-196BAWM-198BAWM-197
	Solanaceae	*Solanum lycopersicum*	'*Ca*. P. australasiaticum subsp. australasiaticum'	20212021	BAWM-206BAWM-207
**Gordonvale**					
	Fabaceae	*Arachis hypogaea*	*'Ca*. P. australasiaticum subsp. ipomoeae'	2020	BAWM-352
**Helidon**					
	Solanaceae	*Solanum lycopersicum*	'*Ca*. P. fabacearum''*Ca*. P. australasiaticum subsp. australasiaticum'	20212021	BAWM-234BAWM-235
**Lyra**					
	Solanaceae	*Capsicum annuum*	'*Ca*. P. australasiaticum subsp. australasiaticum''*Ca*. P. australasiaticum subsp. ipomoeae''*Ca*. P. australasiaticum’ taxon 1	20212021201720212021	BAWM-201BAWM-211a-F3BAWM-060BAWM-238BAWM-237
	Solanaceae	*Solanum lycopersicum*	*'Ca*. P. australasiaticum subsp. australasiaticum'	2017202120212021	BAWM-055BAWM-208BAWM-209BAWM-210
**Mareeba**					
	Asteraceae	*Peripleura diffusa*	*'Ca*. P. australasiaticum subsp. ipomoeae'	2015	BAWM-310
	Asteraceae	*Praxelis clematidea*	*'Ca*. P. australasiaticum subsp. ipomoeae'Mixed infection (*'Ca*. P. fabacearum' and*'Ca*. P. australasiaticum subsp. australasiaticum)	20182018	BAWM-314BAWM-320
	Asteraceae	*Chromolaena odorata*	Mixed infection ('*Ca*. P. fabacearum' and'*Ca*. P. australasiaticum subsp. ipomoeae)	2018	BAWM-316*
	Asteraceae	*Asteraceae* sp.	'*Ca*. P. fabacearum'	2018	BAWM-321*
	Caricaceae	*Carica papaya*	'*Ca*. P. australasiaticum subsp. ipomoeae'	2017	BAWM-309
	Fabaceae	*Cajanus cajan*	'*Ca*. P. fabacearum'	2017	BAWM-323
	Fabaceae	*Crotalaria* sp.	*'Ca*. P. australasiaticum subsp. ipomoeae'	2018	BAWM-315*
	Fabaceae	*Stylosanthes scabra*	'*Ca*. P. australasiaticum' taxon 5'*Ca*. P. fabacearum'Mixed infection (*'Ca*. P. stylosanthis and'*Ca. P*. australasiaticum subsp. ipomoeae')	20182017199819982018	BAWM-324BAWM-322BAWM-252BAWM-255BAWM-326
	Malvaceae	*Sida* sp.	'*Ca*. P. australasiaticum' taxon 4	2018	BAWM-319*
**Severnlea**					
	Solanaceae	*Solanum lycopersicum*	'*Ca*. P. australasiaticum subsp. australasiaticum'	2017	BAWM-056
	Solanaceae	*Solanum* sp.	'*Ca*. P. australasiaticum' taxon 2	2017	BAWM-057*
**Stanthorpe**					
	Apiaceae	*Apium graveolens*	'*Ca*. P. australasiaticum subsp. australasiaticum'	200220022002	BAWM-273BAWM-282BAWM-283
	Apiaceae	*Petroselinum crispum*	'*Ca*. P. australasiaticum subsp. australasiaticum'	2021	BAWM-236-TSE
	Solanaceae	*Capsicum annuum*	'*Ca*. P. australasiaticum subsp. ipomoeae'	2021	7C
**Thorndale**					
	Asteraceae sp.	*Bidens pilosa*	'*Ca*. P. australasiaticum subsp. ipomoeae'	2017	BAWM-063
	Solanaceae	*Solanum lycopersicum*	'*Ca*. P. australasiaticum subsp. australasiaticum'	2017	BAWM-050
	Solanaceae	*Solanum melongena*	'*Ca*. P. australasiaticum subsp. australasiaticum'	2017	BAWM-058
**Walkamin**					
	Fabaceae	*Glycine max*	'*Ca*. P. australasiaticum' taxon 1'*Ca*. P. fabacearum'	20202020	BAWM-150bBAWM-151
	Fabaceae	*Cajanus cajan*	'*Ca*. P. australasiaticum subsp. ipomoeae'	2017	BAWM-311
	Fabaceae	*Crotalaria juncea*	'*Ca*. P. australasiaticum subsp. ipomoeae'	2018	BAWM-312
**Wyberba**					
	Asteraceae	*Osteospermum* sp.	'*Ca*. P. australasiaticum subsp. australasiaticum'	2021	BAWM-215
	Solanaceae	*Petunia* sp.	'*Ca*. P. australasiaticum subsp. australasiaticum'	2021	BAWM-216
**Unknown**					
	Solanaceae	*Capsicum annuum*	'*Ca*. P. australasiaticum subsp. australasiaticum'	2002 2020	BAWM-132BAWM-133BAWM-270
	Solanaceae	*Solanum lycopersicum*	'*Ca*. P. australasiaticum subsp. ipomoeae'	1998	BAWM-250BAWM-251
**VIC**					
**Healesville**					
	Rosaceae	*Fragaria ×Ananassa Duch. Cv. Pajaro*	'*Ca*. P. australasiaticum' taxon 1	2017	BAWM-073
**Kallista**					
	Geraniaceae	*Geranium* sp.	'*Ca*. P. australasiaticum subsp. australasiaticum'	2021	BAWM-246
**Little Hampton**					
	Asteraceae	*Echinacea purpurea*	'*Ca*. P. australasiaticum subsp. australasiaticum'	2020	BAWM-123a
**Melbourne**					
	Solanaceae	*Solanum tuberosum*	'*Ca*. P. australasiaticum subsp. australasiaticum'	2019	4P
**Mildura**					
	Solanaceae	*Solanum lycopersicum*	'*Ca*. P. australasiaticum subsp. australasiaticum'	2019	BAWM-001
	Solanaceae	*Solanum melongena*	'*Ca*. P. australasiaticum subsp. australasiaticum''*Ca*. P. australasiaticum subsp. ipomoeae'	20192019	BAWM-003BAWM-004
	Solanaceae	*Solanum tuberosum*	'*Ca*. P. stylosanthis'	2019	VPRI 43683
	Vitaceae	*Vitis viniferacv*. Chardonnay	'*Ca*. P. australiense'	2020	BAWM-189
**Research**					
	Geraniaceae	*Pelargonium* sp.	'*Ca*. P. australasiaticum subsp. ipomoeae'	2021	BAWM-243
**WA**					
**Carnavon**					
	Convolvulaceae	*Convolvulus clementii*	'*Ca*. P. australasiaticum subsp. australasiaticum'	20202020	BAWM-176BAWM-177
	Cucurbitaceae	*Citrullus lanatus*	'*Ca*. P. australasiaticum subsp. ipomoeae'	2021	BAWM-340
	Cucurbitaceae	*Cucumis sativus*	'*Ca*. P. australasiaticum subsp. ipomoeae'	2020	BAWM-184
	Cucurbitaceae	*Luffa* sp.	'*Ca*. P. australasiaticum subsp. ipomoeae'	2020	BAWM-174
	Fabaceae	*Vigna unguiculata* ssp. *sesquipedalis*	'*Ca*. P. australasiaticum subsp. ipomoeae'	2020	BAWM-179
	Solanaceae	*Capsicum annuum*	'*Ca. P*. australasiaticum subsp. ipomoeae'	202020202021	BAWM-173BAWM-175BAWM-331
	Solanaceae	*Solanum lycopersicum*	'*Ca*. P. australasiaticum subsp. ipomoeae''*Ca. P*. australasiaticum subsp. australasiaticum'	2020202020202020	BAWM-159BAWM-172BAWM-180BAWM-181
	Solanaceae	*Solanum melongena*	'*Ca*. P. australasiaticum subsp. australasiaticum''*Ca*. P. australasiaticum subsp. ipomoeae'	2020202020212021	BAWM-182BAWM-183BAWM-332BAWM-330
	Solanaceae	*Solanum nigrum*	'*Ca*. P. australasiaticum subsp. ipomoeae'	2020	BAWM-178
**Dandaragan**					
	Fabaceae	*Medicago sativa*	'*Ca*. P. fabacearum'Potentially new 16SrII species	202020202020	BAWM-165BAWM-166BAWM-167
	Fabaceae	*Bituminaria bituminosa*	'*Ca*. P. fabacearum'	2019201920192019	BAWM-025BAWM-026BAWM-027BAWM-028
**Kununurra**					
	Apocynaceae	*Catharanthus roseus*	Vigna Little Leaf phytoplasmaMixed infection ('*Ca*. P. stylosanthis' and '*Ca*. P. australasiaticum subsp. ipomoeae)	20212021	BAWM-245BAWM-308
	Caricaceae	*Carica papaya*	*'Ca*. P. australasiaticum subsp. ipomoeae'	20192019	BAWM-041BAWM-042
	Cucurbitaceae	*Cucurbita pepo var. giromontiina* (Zucchini)	*'Ca*. P. australasiaticum subsp. ipomoeae'	20202020	BAWM-187BAWM-188a
	Cucurbitaceae	*Cucurbita maxima* (Pumpkin)	'*Ca*. P. australasiaticum subsp. ipomoeae'Mixed infection ('*Ca*. P. fabacearum' and '*Ca*. P. australasiaticum subsp. australasiaticum)	20202021	BAWM-333BAWM-186
	Fabaceae	*Cicer arietinum*	'*Ca*. P. fabacearum'	2019	BAWM-043
	Fabaceae	*Crotalaria juncea*	*'Ca*. P. australasiaticum subsp. ipomoeae'	2019	BAWM-046
	Fabaceae	*Glycine max*	Mixed infection ('*Ca*. P. fabacearum' and '*Ca*. P. australasiaticum subsp. australasiaticum)	2019	BAWM-044
	Fabaceae	*Vigna radiata*	*'Ca*. P. australasiaticum subsp. ipomoeae'	2019	BAWM-045
	Fabaceae	*Cajanus cajan*	'*Ca*. P. australasiaticum subsp. ipomoeae'	2020	BAWM-185
	Goodeniaceae	*Goodenia scaevolina*	Mixed infection ('*Ca*. P. stylosanthis' and'*Ca*. Phytoplasma australasiaticum subsp. australasiaticum)	2021	BAWM-307
	Myrtaceae	*Melaleuca* sp.	'*Ca*. P. melaleucae'	2022	BAWM-354A
	Solanaceae	*Solanum lycopersicum*	*'Ca*. P. australasiaticum subsp. ipomoeae'	2003	BAWM-284
	Solanaceae	*Solanum* sp.	*'Ca*. P. australasiaticum subsp. australasiaticum'	2004	BAWM-285
**Lancelin**					
	Solanaceae	*Solanum nigrum*	*'Ca*. P. australasiaticum subsp. ipomoeae'	2021	BAWM-328
**Serpentine**					
	Malvaceae	*Gomphocarpus fruticosus*	*'Ca*. P. australasiaticum subsp. australasiaticum'	202120212021	BAWM-212BAWM-213BAWM-214

* An asterisk (*) in the sample name(s) column indicates samples for which the host identity was determined using DNA barcode analysis.

Phytoplasma samples analysed in this study were collected from all the states and territories of Australia, apart from the Australian Capital Territory (ACT), South Australia (SA), and Tasmania (TAS) (Tables 1 and S1). No samples were collected between 2019 and 2022 in SA due to Covid-19 travel restrictions. There was also an absence of plants showing typical phytoplasma symptoms in TAS during the 2019–2022 collection period (Callum R. Wilson, personal communication), which corresponds to previous observations of low phytoplasma prevalence for the state [54].

An asterisk (*) in the sample name(s) column indicates samples for which the host identity was determined using DNA barcode analysis.

### blastn of the 16S rRNA gene for phytoplasma-positive samples

Based on blastn analysis of the 16S rRNA gene PCR amplicons (Table 2 and S1), samples had single top hits with ten different phytoplasma taxa, including ‘*Ca*. Phytoplasma australasiaticum subspecies australasiaticum’ (*n*=91), ‘*Ca*. Phytoplasma australasiaticum subspecies ipomoeae’ (*n*=69), ‘*Ca*. Phytoplasma fabacearum’ (*n*=15), ‘*Ca*. Phytoplasma bonamiae’ (*n*=2), ‘*Ca*. Phytoplasma planchoniae’ (*n*=1, BAWM-156b), ‘*Ca*. Phytoplasma stylosanthis’ (*n*=6), ‘*Ca*. Phytoplasma melaleucae’ (*n*=2), ‘*Ca*. Phytoplasma australiense’ (*n*=1, BAWM-189), ViLL phytoplasma (*n*=3, BAWM-245 shared a lower nucleotide identity than BAWM-336 and BAWM-337 with the strain at the NCBI), and *Waltheria* Little Leaf phytoplasma (WaLL, *n*=1, BAWM-227). Cloning and Sanger sequencing of the 16S rRNA gene PCR amplicon done for samples BAWM-252, BAWM-253, BAWM-255, and BAWM-257 identified the presence of mixed phytoplasma infections of ‘*Ca*. Phytoplasma australasiaticum subspecies ipomoeae’ and ‘*Ca*. Phytoplasma stylosanthis’ for each sample (Tables 2 and S1). All of these phytoplasma taxa identified based on blastn analyses in this study have been previously detected and reported in Australia based on 16S rRNA-based sequence analysis [12,15,18, 26, 55, 56]

**Table 2.** Summary of blastn top hits of the 16S rRNA gene sequences obtained for the samples investigated in this study, including the putative phytoplasma taxon identified, the number of samples with this result, and the range of percent of nucleotide identities shared with the top hit

‘*Candidatus* Phytoplasma’ top hits	No. samples	Range of nucleotide identities with the top hit (%)
**‘*Ca.* Phytoplasma australasiaticum subsp. australasiaticum’**	91	98.88–100 %
**‘*Ca.* Phytoplasma australasiaticum subspecies ipomoeae’**	69	98.95–100 %
**‘*Ca.* Phytoplasma fabacearum’**	15	100 %
**‘*Ca.* Phytoplasma bonamiae’**	2	100 %
**‘*Ca.* Phytoplasma planchoniae’**	1	100 %
**‘*Ca.* Phytoplasma stylosanthis’**	6	100 %
**‘*Ca.* Phytoplasma melaleucae’**	2	100 %
**‘*Ca.* Phytoplasma australiense’**	1	99.90 %
***Vigna* Little Leaf (ViLL) phytoplasma**	3	99.83–99.92 %
*Waltheria* Little Leaf (WaLL) phytoplasma*	1	na
Mixed phytoplasma infection:**‘*Ca.* Phytoplasma australasiaticum subspecies ipomoeae’ and ‘*Ca.* Phytoplasma stylosanthis’†**	4	100 %100 %

* The previous identification of BAWM-227 as a WaLL phytoplasma based on RFLP in the nt DITT phytoplasma database was used as no full -length 16S rRNA gene exists for this taxon [13]. However, the 16S rRNA gene of this phytoplasma shared 99.65 % nucleotide identity with that of ‘*Ca*. Phytoplasma asiaticum’.

† Mixed infection was identified for these samples by Sanger sequencing cloned 16S rRNA PCR amplicons.

### Metagenomic sequence data outputs and metagenome-based host identifications

#### Metagenomic sequencing output and phytoplasma genome information

After metagenomic HTS, 178 of the 195 total samples passed the phytoplasma genome criteria for further comparative genomic analyses in this study (Table S1). The Illumina sequence data output for these 178 samples ranged from 0.31 Gb (sample BAWM-193a-F1) to 32.60 Gb (sample BAWM-354A) with an average output of 4.76 Gb per metagenomic library (Table S1). The phytoplasma genome sizes of these 178 samples ranged between 321 651 bp (sample BAWM-201) and 1 488 020 bp (sample BAWM-255), with an average genome size of 632,634 bp for of all 178 phytoplasma samples (Table S1). Of the 178 draft genomes, an average of 28 tRNA gene sequences were recovered per genome and ranged between 13 tRNA genes (sample BAWM-198) to 61 tRNA genes (sample BAWM-255) (Table S1). The most tRNA genes recovered from a complete phytoplasma genome to date is 35 from the genome of ‘*Ca*. Phytoplasma australiense’ strain NZSb11 [57], indicating that the phytoplasma genome data of 13 of the 178 samples that had more than 35 tRNA genes annotated may represent tRNAs of more than one phytoplasma species in the sample (Table S1). An average of two phytoplasma rRNA genes could be annotated from these 13 genomes, with a max of five rRNA genes (sample BAWM-307) and none obtained from sample BAWM-350 (Table S1). To date, two identical or nearly identical 16S rRNA genes are known to be encoded per phytoplasma genome [1,36] indicating that sample BAWM-307 potentially harbours a mixed phytoplasma infection.

Phytoplasma genome sequences that were <300 000 bp were recovered for 17 samples in six different host families (Table S1). These genome sequences ranged in size from 1784 bp (sample BAWM-004) to 289 060 bp (sample BAWM-184), with an average size of 114 181 bp (Table S1). The average data output for these 17 samples was 5.07 Gb, with a range of 1.28 Gb (sample BAWM-173) to 22.53 Gb (sample BAWM-189) (Table S1). An average of six tRNA genes could be retrieved from these 17 phytoplasma genomes (range 0 tRNA genes for samples BAWM-003, BAWM-004, BAWM-083, BAWM-216, and BAWM-233 to 21 tRNA genes for BAWM-183). No rRNA genes were retrieved from seven of the 17 phytoplasma genomes. These samples were not used in further genomic-based analyses as these results indicate poor-quality genomes from which limited information can be obtained, including the 16S rRNA gene-based correlation of taxon identification prior to and after metagenomic HTS [58].

#### Phytoplasma taxon identification using whole-genome comparisons using ANI estimates and 16S rRNA gene analyses

##### Subspecies of ‘*Ca*. Phytoplasma australasiaticum’

**Fig. 1.**
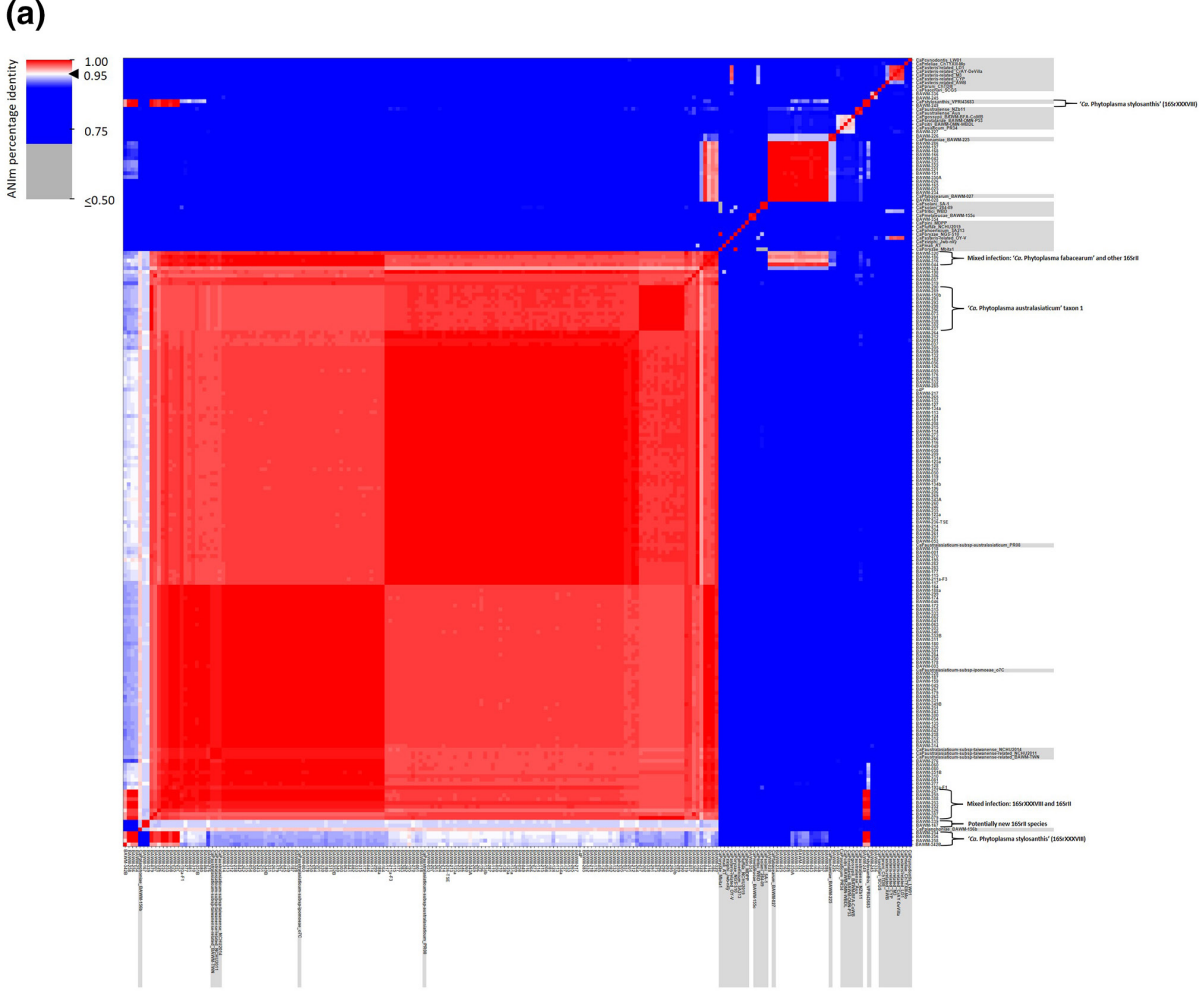

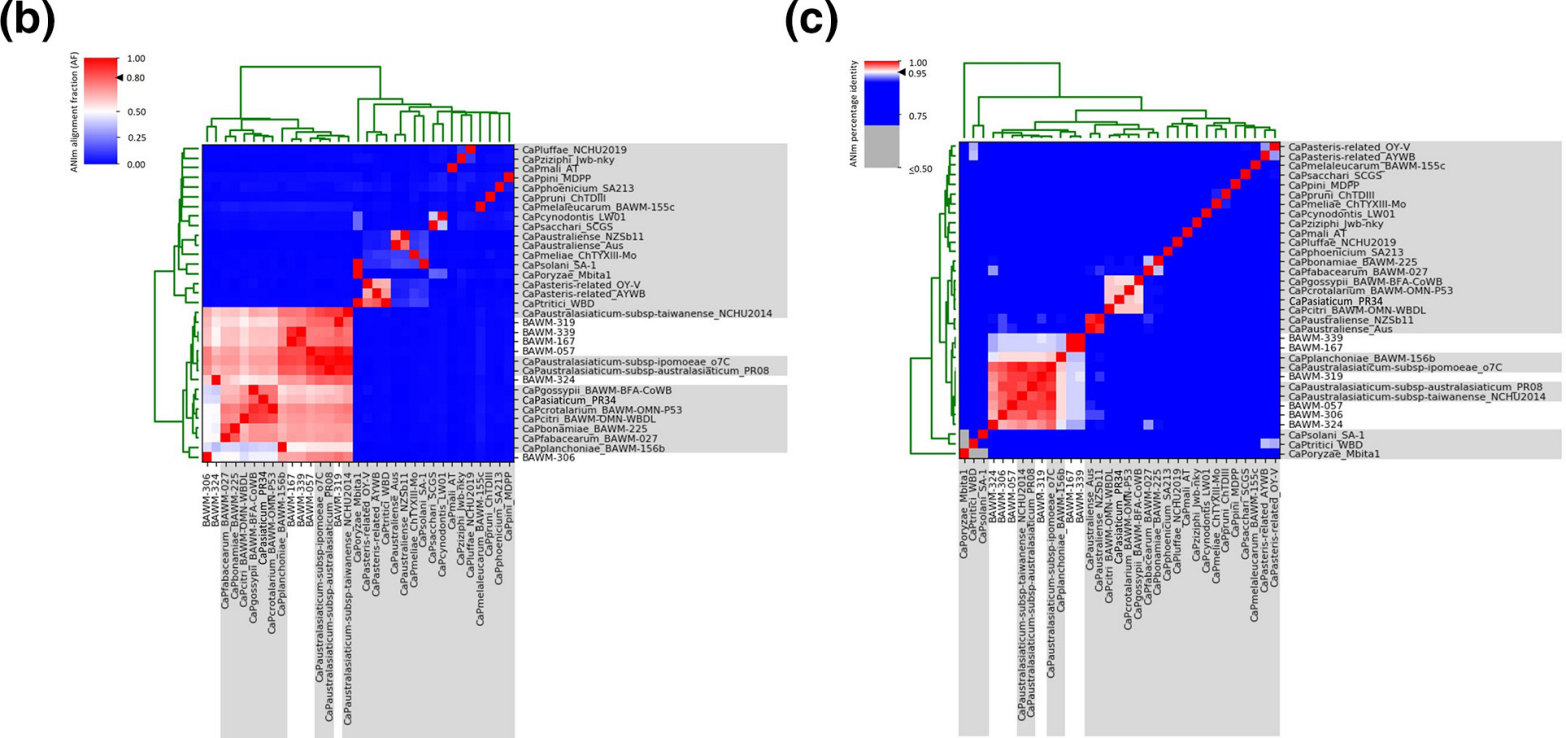
Whole-genome comparisons for phytoplasma genome sequence data obtained for 178 samples. (**a**) ANI heatmap, generated by pyani version 0.2.10 using the ANIm algorithm, for all strains sequenced in this study alongside representative and publicly available genomes. Some clusters are highlighted using brackets. (**b**) ANI percentages and (**c**) alignment fractions (AF) in each pairwise comparison of samples that did not cluster with representative genomes in Fig. 1a. The genomes of representative strains and publicly available are shaded in grey. See colour gradient representing the percent identities in the heatmaps of (**a**) and (**b**) or the AF per genome in (**c**).

Based on whole-genome ANI analyses (Fig. 1; Table S1), 160 of the 178 samples (ca. 90 %) used in further genome-based analyses clustered at >96 % ANI solely with representative genome sequences of 16SrII phytoplasmas. The majority of these samples classified within the 16SrII phytoplasma group were identified as ‘*Ca*. Phytoplasma australasiaticum’ subspecies, including of ‘*Ca*. Phytoplasma australasiaticum subsp. australasiaticum’ (*n*=67), ‘*Ca*. Phytoplasma australasiaticum subsp. ipomoeae’ (*n*=51), and strains identified as a new ‘*Ca*. Phytoplasma australasiaticum’ subspecies (*n*=12, referred to as ‘*Ca*. Phytoplasma australasiaticum’ taxon 1) (Fig. 1).

**Fig. 2.**
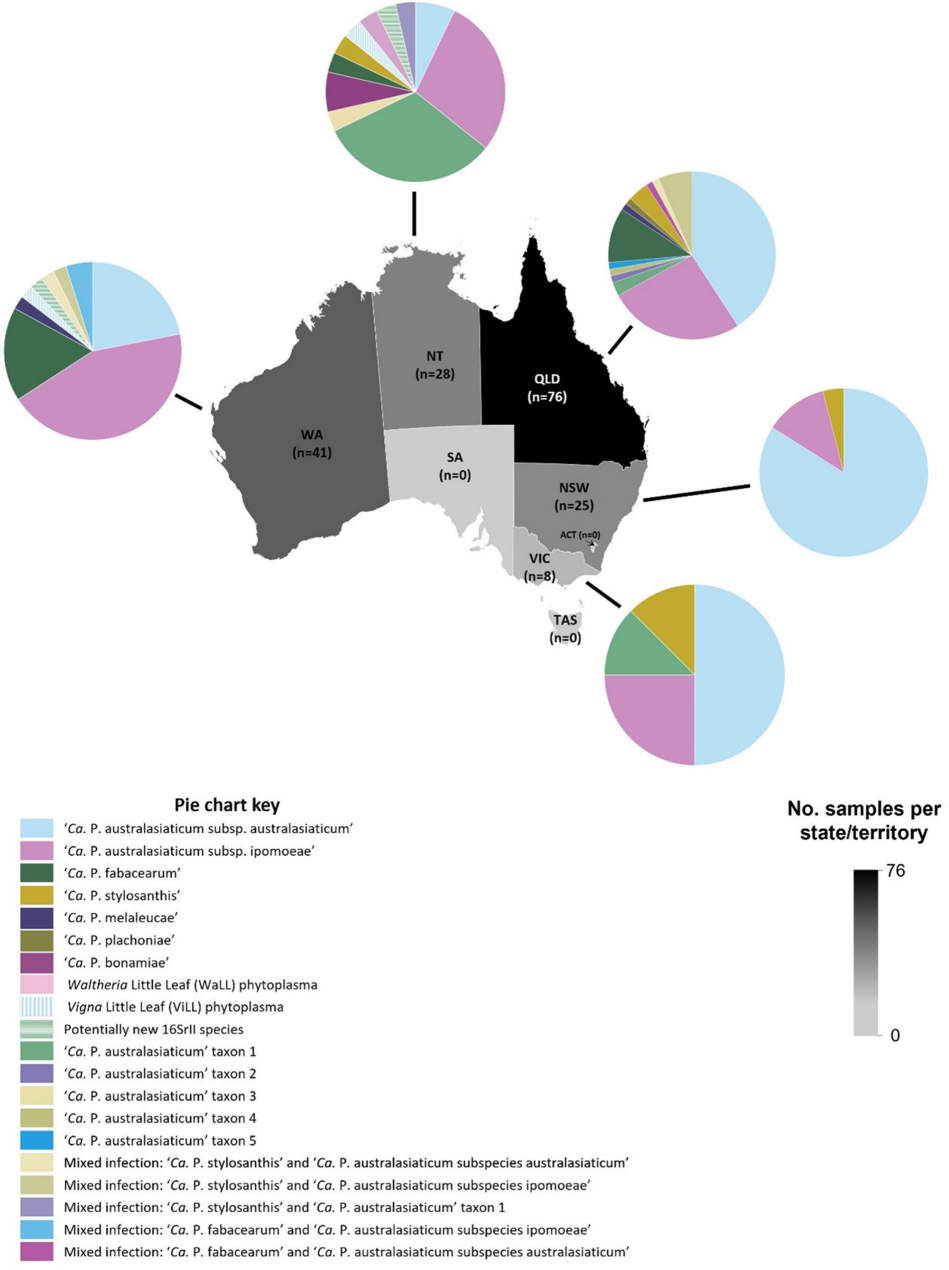
Map of Australia showing the number of phytoplasma-positive samples collected per state or territory, with pie charts illustrating the proportions of ANI identified phytoplasma taxa identified per state or territory (see key below for descriptions of colour-coding). The scale on the right indicates the number of samples collected for each state or territory, with the number in brackets indicating the total number of ANI-identified samples per location within the map area. Abbreviations: ACT, Australian Capital Territory; NSW, New South Wales; NT, Northern Territory; QLD, Queensland; TAS, Tasmania; VIC, Victoria; WA, Western Australia.

When the 12 16S rRNA sequences of the ‘*Ca*. Phytoplasma australasiaticum’ taxon 1 samples extracted from the genomic sequences were queried further, the historic samples were classified as ‘TBB’ (i.e. ‘*Ca*. Phytoplasma australasiaticum’) in the NT DITT phytoplasma database and their 16S rRNA genes shared the highest nucleotide sequence similarity and coverage with the ‘*Ca*. Phytoplasma australasiaticum subsp. australasiaticum’ representative strain PR08 (99.92 % nucleotide sequence identity, 100 % coverage) in blastn analyses (Table 1). Additionally, these sequences could only be differentiated from ‘*Ca*. Phytoplasma australasiaticum subsp. australasiaticum’ and ‘*Ca*. Phytoplasma australasiaticum subsp. ipomoeae’ during *in silico* RFLP analysis by the *Hae*III restriction enzyme (Fig. S1). Therefore, the identification and subsequent characterization of this ‘*Ca*. Phytoplasma australasiaticum’ subspecies was likely missed in previous analyses as the *Hae*III restriction enzyme was infrequently used during *in vitro* RFLP analyses [13,14, 16, 59]. These results illustrate the low resolution of the RFLP of the 16S rRNA gene sequence to delimit separate phytoplasma taxa compared to species and subspecies characterization that is possible using the genome ANI, which has also been emphasized in previous studies [36].

The phytoplasma genomes obtained from samples BAWM-057, BAWM-306, and BAWM-319 formed their own clusters in the ANI analyses and do not share 100 % ANI with any other sample. However, these three genomes all clustered with the ‘*Ca*. Phytoplasma australasiaticum’ species at <100 % ANI (Fig. 1b) with approximately 80 % AF (Fig. 1c). These ANI results suggest that each of these samples might represent three novel subspecies (referred to as ‘*Ca*. Phytoplasma australasiaticum’ taxon 2, 3, and 4, respectively). Both BAWM-057 and BAWM-319 were indistinguishable from ‘*Ca*. Phytoplasma australasiaticum subsp. australasiaticum’ based on nucleotide identities of their 16S rRNA gene in the blastn analyses (Table S1). The 16S rRNA gene sequence of BAWM-306 shared the highest nucleotide identity with ‘*Ca*. Phytoplasma australasiaticum subsp. australasiaticum’ (99.75 % identity, 100 % coverage, 4404 total score) (Table S1).

The phytoplasma genome obtained from sample BAWM-324 shares the lowest ANI and AF values with other with ‘*Ca*. Phytoplasma australasiaticum’ genomes analysed in this study (ca. 96 % ANI and <80 % AF) but is still grouped closely with species in the ‘*Ca*. Phytoplasma australasiaticum’ cluster. These results suggest that BAWM-324 might represent a single new ‘*Ca*. Phytoplasma australasiaticum’ subspecies (Fig. 1b, c; referred to as ‘*Ca*. Phytoplasma australasiaticum’ taxon 5). Sequence divergence between the 16S rRNA gene sequences of BAWM-324 and its top hit, ‘*Ca*. Phytoplasma australasiaticum subsp. ipomoeae’, was also observed (<100 % nucleotide identity, 100 % coverage, 2207 total score; Table S1), which supports these ANI results.

Together, these results suggest that the previously unrecorded taxa, ‘*Ca*. Phytoplasma australasiaticum’ taxon 1 to taxon 5, are endemic to Australia. This is supported by ‘*Ca*. Phytoplasma australasiaticum’ taxon 1 being present, but misclassified, in a historic sample collected in 2004 (Table S1) but also because these phytoplasma taxa have not been detected in any other country to date based on the 16S rRNA sequences and the limited number of 16SrII phytoplasma genomes that are publicly available. Using the genome-sequence data obtained for these strains in this study, further analyses are required and could be done to confirm whether these five new taxa are truly distinct subspecies of ‘*Ca*. Phytoplasma australasiaticum’ and not artefacts generated during metagenomic sequencing and analyses [36,37].

##### A putatively new species within the 16SrII phytoplasma group

The phytoplasma strains obtained from samples BAWM-167 and BAWM-339 shared 100 % ANI and >80 % AF with each other and approximately 94 % ANI and <80 % AF with any phytoplasma genomes used in this study, including the closely related subspecies of ‘*Ca*. Phytoplasma australasiaticum’ (Fig. 1b, c). These results indicate that the phytoplasma strains from samples BAWM-167 and BAWM-339 may represent a novel ‘*Ca*. Phytoplasma’ species within the 16SrII group. The 16S rRNA gene analyses support the divergence of these two strains compared to other previously described 16SrII phytoplasmas. The 16S rRNA genes of BAWM-167 and BAWM-339 had 99.92 % sequence similarity to the reference sequence of ‘*Ca*. Phytoplasma australasiaticum subsp. australasiaticum’ (100 % coverage, 2180–2211 total score) (Table S1). Further phylogenomic analyses are required to confirm whether these three taxa are truly distinct subspecies and not due to artefacts generated during metagenomic sequencing and analyses. However, the identification of this putative novel species from two distinct hosts (*Medicago sativa* and *Ipomoea* sp.) across a large geographic separation may provide positive support for the existence of this species.

This species could be endemic to Australia because these two detections are the first time this species has been detected globally based on the 16S rRNA sequences and they were detected in two geographically distinct areas in Australia (Darwin, NT and Dandaragan, WA) in different hosts and years.

##### Other phytoplasma taxa identified in this study, which have previously been recorded in Australia

Strains of the 16SrII species, ‘*Ca*. Phytoplasma fabacearum’, formed the third largest cluster (*n*=16) in the ANI analyses, while the cluster containing the group 16SrXXXVIII species ‘*Ca*. Phytoplasma stylosanthis’ was the fifth largest (*n*=6) (Fig. 1). The single WaLL phytoplasma (group 16SrII) strain from BAWM-227, showed between 90 and 95 % ANI with four 16SrII phytoplasma species previously determined to be closely related to each other [37], namely ‘*Ca*. Phytoplasma citri’, ‘*Ca*. Phytoplasma asiaticum’, ‘*Ca*. Phytoplasma gossypii’, and ‘*Ca*. Phytoplasma crotalariae’ (Fig. 1). The two ‘*Ca*. Phytoplasma bonamiae’ strains (group 16SrII) formed their own cluster with 100 % ANI between them in the pairwise analysis and shared the next highest ANI with strains of ‘*Ca*. Phytoplasma fabacearum’ (ANI of <94 %, Fig. 1). The two ‘*Ca*. Phytoplasma melaleucae’ strains (group 16SrXV) formed their own cluster with 100 % ANI between them in the pairwise analysis (Fig. 1). The ViLL phytoplasma strains (16 Sr group unassigned) clustered with each other, but with a pairwise ANI of ca. 97 % to each other.

The blastn analyses of the 16S rRNA genes of all these taxa supported the ANI results (Fig. S1A), and the WaLL phytoplasma was characterized based on RFLP in previous analyses (NT DITT record; Table 2) [13]. Further, the ANI results of WaLL and ViLL phytoplasmas suggest that these taxa could be described as two novel ‘*Ca*. Phytoplasma’ species (ANI <95 % with any other genome available for described phytoplasma species). Future work is required to determine whether the WaLL and ViLL phytoplasmas meet the updated requirements for the description of novel ‘*Ca*. Phytoplasma’ species [31] and whether the two ViLL strains represent two individual subspecies. Additionally, the competent insect vector species of WaLL and ViLL taxon remain to be determined.

##### The identification of mixed phytoplasma infections

Close analysis of the ANI heat map revealed evidence of mixed phytoplasma infections (Fig. 1), where several samples showed a high ANI with two representative genomes. Samples BAWM-044, BAWM-186, and BAWM-316 had mixed infections comprising of ‘*Ca*. Phytoplasma fabacearum’ and ‘*Ca*. Phytoplasma australasiaticum subsp. ipomoeae’. blastn analyses of the 16S rRNA genes obtained for these samples identified the presence of ‘*Ca*. Phytoplasma australasiaticum subsp. ipomoeae’ but failed to identify ‘*Ca*. Phytoplasma fabacearum’ (Table S1).

Sample BAWM-320 had a mixed infection of ‘*Ca*. Phytoplasma fabacearum’ and ‘*Ca*. Phytoplasma australasiaticum subsp. australasiaticum’ based on the ANI analyses (Fig. 1). However, only ‘*Ca*. Phytoplasma fabacearum’ was identified from this sample based on blastn of the 16S rRNA gene obtained (Table S1).

Six samples had mixed infections of ‘*Ca*. Phytoplasma stylosanthis’ and ‘*Ca*. Phytoplasma australasiaticum subsp. ipomoeae’ based on the ANI analysis (samples BAWM-252, BAWM-253, BAWM-255, BAWM-257, BAWM-308, BAWM-326; Fig. 1). The 16S rRNA gene of ‘*Ca*. Phytoplasma stylosanthis’ was obtained for all of these samples upon Sanger sequencing of the PCR amplicon apart from BAWM-308, which was identified as ‘*Ca*. Phytoplasma ipomoeae’ when the PCR amplicon was Sanger sequenced (Table S1). Sanger sequencing of the cloned 16S rRNA gene PCR amplicon of samples BAWM-252, BAWM-253, BAWM-255, and BAWM-257 confirmed these mixed infections (data not shown).

‘*Ca*. Phytoplasma stylosanthis’ was identified in a mixed infection with either ‘*Ca*. Phytoplasma australasiaticum’ taxon 1 (sample BAWM-079) or with ‘*Ca*. Phytoplasma australasiaticum subsp. australasiaticum’ (sample BAWM-307). After PCR amplification and Sanger sequencing, only the 16SrII taxon was detected for these samples (Table S1).

When revisiting the number of tRNA gene sequences annotated from these 12 samples identified to contain mixed phytoplasma infections, more than 35 tRNA gene sequences were identified from all of these samples apart from sample BAWM-307 from which 32 tRNA genes were obtained (Table S1). Further, only one sample for which no mixed phytoplasma infection was identified using the ANI approach encoded more than 35 tRNA genes (BAWM-311, *n*=40 tRNA genes). These results highlight that the metagenomic sequencing, assembly, and tRNA annotation approach used in this study can sufficiently resolve the distinct tRNA genes encoded by each phytoplasma species and, thus, support the utility of using the tRNA count as an indicator of mixed phytoplasma infections in a sample in addition to genome completeness criteria that has been proposed previously [60,61].

The results of species identification based on the 16S rRNA gene and whole-genome comparisons emphasize several important implications of these approaches to phytoplasma identification. Firstly, the 16S rRNA gene sequences obtained by either method often only represented one of the phytoplasma taxa involved in the mixed infection. It is likely that this arose due to differences in the titres of the multiple phytoplasma taxa in the sample, with only the one gene sequence being obtained by direct Sanger sequencing or more genomic sequence data obtained from the phytoplasma present at the higher titre. Alternatively, the multiple 16S rRNA gene sequences of closely related phytoplasma taxa obtained in a sample may have been missed during the process of obtaining the consensus sequence from both Sanger sequencing and metagenomic HTS data.

##### Phytoplasma identifications made for samples that could not be used in ANI analyses

For the samples for which insufficient phytoplasma data was obtained for ANI analyses, the 16S rRNA sequences shared high sequence similarity with ‘*Ca*. Phytoplasma australasiaticum subsp. australasiaticum’ (*n*=9 sequences), ‘*Ca*. Phytoplasma australasiaticum subsp. ipomoeae’ (*n*=7 sequences), ‘*Ca*. Phytoplasma australiense’ (*n*=1 sequence, BAWM-189), and the ViLL phytoplasma (*n*=1, BAWM-337) (Table S1). These results highlight the pitfall of applying metagenomic-based approaches to identify the species diversity of phytoplasmas obtained from a diversity of host species. Specifically, it may be difficult to get sufficient data from hosts that harbour low titre infections for comparison with other taxa from metagenome sequencing. Whereas PCR of the 16S rRNA gene enriches for these regions of interest that provide taxonomically informative information [62], albeit at a low taxonomic resolution [37]. This highlights the need to sequence additional genomic regions in diversity studies, and/or to have pre-sequencing enrichment tools for phytoplasma cells or DNA to improve genome sequence retrieval and assembly for genomic analysis relevant for applications such as taxonomy [38,63,65]

### Host and geographic ranges of the phytoplasma taxa identified in this study

#### Identification of unknown hosts

Eight weed samples were either unknown or not confidently identified to family, genus or species based on visual identification. The combination of two barcodes, *mat*K and *rbc*L, extracted from metagenomic data as well as occurrences of the identified species in the geographic region of collection, as recorded in the Australasian Virtual Herbarium, were used to indicate the host plant species (Table 3).

**Table 3.** Summary of samples for which initial host identifications were unresolved to the family-, genus- or species-level based on visual inspections and for which additional gene regions obtained from metagenomic data were used to determine the host identity. The genes used for plant host identifications included the *maturase K* (*mat*K) and *ribulose-bisphosphate carboxylase* (*rbc*L) genes, and the *cytochrome C oxidase subunit 1* (*co*I) gene was used for insect identification. The e-value, percent identity, and bitscores of the top blastn hit(s) for the sample are provided to illustrate the support for the gene-based host identification. na=Not applicable

			Top hit name (accession no.), e-value, nucleotide percent identity (%), bitscore per barcode		
Sample name	Host identification based on morphology	Host identification based on barcode analysis	*mat*K	*rbc*L	*co*I
BAWM-037	Unknown weed	Family: *Malvaceae*Genus: *Sida*Species: *Sida* sp.	*Sida rhombifolia* (MN006709);*Sida acuta* (MN006706)1.98E-38, 100 %, 22	*Sida cordifolia* (MH588540)8.18E-47, 100 %, 28	na
BAWM-057	Unknown weed	Family: *Solanaceae*Genus: *Solanum*Species: *Solanum* sp.	*Solanum chenopodioides* (MH464378);*Solanum americanum* (MK244345)1.72E-39, 100 %, 32	*Solanum tripartitum* (KF546075); *Solanum palitans* (KF546070)4.63E-75, 97.1 %, 57	na
BAWM-079	Unknown weed	Family: *Solanaceae*Genus: *Solanum*Species: *Solanum* sp.	*Solanum nigrum* (KC535797)1.59E-35, 91.0 %, 22	*Solanum rostratum* (NC_057245)1.00E-69, 100 %, 345	na
BAWM-302	Unknown weed	Family: *Solanaceae*Genus: *Solanum*Species: *Solanum* sp.	*Solanum elaeagnifolium* (EU983576);*Solanum melanospermum* (EU983565)0, 96.4 %, 100	*Solanum rostratum* (MK526696)0, 95.4 %, 100	na
BAWM-315	Family: *Fabaceae*	Family: *Fabaceae*Genus: *Crotalaria*Species: *Crotalaria* sp.	*Crotalaria juncea* (JQ619982.1)3.6E-41, 98.90 %, 163	*Crotalaria sagittalis* (KY584333); *Crotalaria spectabilis* (KJ773415); *Crotalaria pallida* (KJ773413)7.6E-29, 100 %, 13	na
BAWM-316	Family: *Lamiaceae*	Family: *Asteraceae*Species: *Chromolaena odorata*	*Chromolaena odorata* (MN558588)5.6E-57, 100 %, 45	*Chromolaena odorata* (MH767490)0, 91.9 %, 58	na
BAWM-319	Family: *Lamiaceae*	Family: *Malvaceae*Genus: *Sida*Species: *Sida* sp.	*Sida rhombifolia* (KT966997);*Sida fallax* (MF350256)3.00E-46, 94.78 %, 180	*Sida rhombifolia* (MH549993)1.03E-50, 100 %, 30	na
BAWM-321	Family: *Lamiaceae*	Family: *Asteraceae*Genus: UndeterminedSpecies: Undetermined	*Praxelis clematidea* (KX526581)2.58E-176, 94.8 %, 100*Chromolaena odorata* (MN558588)5.59E-173, 94.3 %, 100	*Chromolaena odorata* (KY986097)1.83E-62, 96.7 %, 14	na
BAWM-342B	Genus: *Orosius*	Species: *Orosius argentatus*	na	na	*Orosius argentatus* (KR030333)0, 99.6 %, 5
BAWM-343A	Genus: *Orosius*	Species: *Orosius argentatus*	na	na	*Orosius argentatus* (KR030333)0, 99.9 %, 5

Two plants visually identified as members of the *Lamiaceae* were identified as *Asteraceae* members, including *Chromolaena odorata* (BAWM-316) and an undetermined species likely in the *Chromolaena* or *Praxelis* genera (BAWM-321). (Tables 3 and S1). One weed species (sample BAWM-319) was also visually identified as a member of the *Lamiaceae* but was identified as a member of the Malvaceae instead based on the DNA barcodes (*Sida* sp., BAWM-319) (Table S1). The host species of BAWM-319 is likely *Sida rhombifolia* as this species is present in QLD where the sample was collected, whereas *Sida fallax* reports were made from WA in the Australasian Virtual Herbarium records. Species in the *Sida* genus have been reported as a host in Australia previously [12]. The results for these three samples are consistent with other phytoplasma detections made in Australia, where members in the *Lamiaceae* are not known to host phytoplasmas in Australia but where several *Asteraceae* and *Malvaceae* species have been recorded [27].

Samples BAWM-037, BAWM-057, BAWM-079, and BAWM-302 were all recorded as ‘unknown weed’ species based on morphological observations (Tables 3 and S1). Samples BAWM-057 and BAWM-302 were subsequently identified to the genus-level based on BLASTn of the *mat*K and *rbc*L gene sequences (both *Solanum* spp., Solanaceae). The host of BAWM-079 was also identified as a *Solanum* sp. (Solanaceae) by the host DNA barcode analyses (Table 3, either *Solanum nigrum* or *Solanum rostratum*). However, it is likely that the host species of BAWM-079 is *Solanum nigrum* as this plant species has a wide geographic distribution in Australia based on the Australasian Virtual Herbarium, including in the NT where BAWM-079 was sampled, and due to the high blastn results for this gene with *Solanum nigrum* (228 total score, 5E-60 e-value, 99.2 % identity; GenBank accession number: M588530). *Solanum rostratum* is not present in the NT according to the Australasian Virtual Herbarium. However, the *rbc*L gene sequence had higher blastn scores with *Solanum rostratum* and it was, therefore, recorded in Table 3. Based on blastn of the *mat*K and *rbc*L genes obtained from the metagenomic data, the host of BAWM-037 was identified as a *Sida* sp. (Malvaceae). The host species of BAWM-037 could not be determined due to the inconsistencies between the top hit species listed for the two DNA barcodes (Table 3).

Two insect hosts were identified as *Orosius* sp. based on their external morphology (samples BAWM-342B and BAWM-343A). Using sequence analyses of their *coI* gene, both samples were identified as *Orosius argentatus*. Based on several studies, *Orosius argentatus* is a known phytoplasma vector in Australia and is detected across a broad geographic range in Australia [9,29, 30, 66].

These sequence-supported identifications of plant or insect hosts at the species-, genus-, or family-level when they were not known based on visual inspection highlight the added benefit of a metagenomic-based approach to investigating phytoplasma diversity and their host associations. However, the host species listed using this approach are considered preliminary indications of the host taxa sampled, especially when (i) the nucleotide identities of the DNA barcodes were not identical to those of voucher specimens on the NCBI, despite the nucleotide identities being above 90 % in all cases in this study (Table 3), (ii) recording species-level identifications, and (iii) considering that some barcodes may be missing for the species under investigation but for which they are available for a closely related species [67,68]. This is due to the limitations of the available and well-validated plant DNA barcodes in the public databases.

#### Summary of phytoplasma plant and insect hosts for Australia

Over 40 different insect or plant genera were reported as phytoplasma hosts in this study, representing 16 plant families and one insect family (*Cicadellidae*) (Table 4) (Tables1^ 4^ and S1). A total of 56 unique species were sampled. Of the 195 phytoplasma-infected samples, 158 were classed as crop species, 24 as weed species, seven as ornamental plants, six were native plants, and three were individual insect samples (Tables 4 and S1). Of the 158 crop species, ca. 59 % were in the *Solanaceae* (*n*=91), ca. 17 % were in the *Fabaceae* (*n*=34), ca. 8 % were in the *Cucurbitaceae* (*n*=15), with the remaining samples from the *Apiaceae* (*n*=4), *Caricaceae* (*n*=4), *Convolvulaceae* (*n*=3), *Vitaceae* (*n*=2), and *Asteraceae* (*n*=1) families. The crop species *Solanum lycopersicum* (*n*=42), *Capsicum annuum* (*n*=33), and *Solanum melanogena* (*n*=16), and *Stylosanthes scabra* (*n*=10) were collected in high numbers (Tables 1 and S1).

**Table 4.** List of phytoplasma hosts investigated in this study, characterized based on whether the host was recorded previously as a phytoplasma host/putative vector or not, and whether these hosts were classified as crop (C), insects (I), native plant (NP), ornamental (O), or weed (W) in this study. A list of the phytoplasma taxa that were identified are listed for the respective host

Host family	Host genus/species name	**Host classification**	Phytoplasma taxa detected
**Previously recorded phytoplasma hosts for Australia collected in this study**			
*Apiaceae*	*Apium graveolens*	C	'*Ca*. P. australasiaticum subsp. australasiaticum'
*Apocynaceae*	*Catharanthus roseus*	O	*Vigna* Little Leaf phytoplasma;Mixed infection ('*Ca*. P. australasiaticum subsp. ipomoeae' and '*Ca*. P. stylosanthis')
*Asteraceae* sp.	*Asteraceae* sp.*	W	'*Ca*. P. fabacearum'
*Caricaceae*	*Carica papaya*	C	'*Ca*. P. australasiaticum subsp. ipomoeae';'*Ca*. P. stylosanthis'
*Cicadellidae*	Orosius argentatus**	I	'*Ca*. P. australasiaticum subsp. australasiaticum';'*Ca*. P. stylosanthis'
*Cicadellidae*	*Orosius orientalis*	I	'*Ca*. P. australasiaticum subsp. ipomoeae' (blastn)
*Convolvulaceae*	*Bonamia pannosa*	W	'*Ca*. P. bonamiae'
*Convolvulaceae*	*Ipomoea batatas*	C	'*Ca*. P. australasiaticum subsp. australasiaticum'
*Convolvulaceae*	*Ipomoea* sp.	W	New 16SrII species
*Cucurbitaceae*	*Cucurbita maxima* (Pumpkin)	C	'*Ca*. P. australasiaticum subsp. ipomoeae';Mixed infection ('*Ca*. P. australasiaticum subsp. ipomoeae' and '*Ca*. P. fabacearum')
*Fabaceae*	*Arachis hypogaea*	C	'*Ca*. P. australasiaticum subsp. ipomoeae'
*Fabaceae*	*Cajanus cajan*	C	'*Ca*. P. australasiaticum subsp. ipomoeae'
*Fabaceae*	*Cicer arietinum*	C	'*Ca*. P. fabacearum'
*Fabaceae*	*Crotalaria* sp.	W	'*Ca*. P. australasiaticum subsp. ipomoeae'
*Fabaceae*	*Crotalaria* sp.‡	W	'*Ca*. P. australasiaticum subsp. ipomoeae'
*Fabaceae*	*Glycine max*	C	'*Ca*. P. australasiaticum' taxon 1;'*Ca*. P. fabacearum';Mixed infection ('*Ca*. P. australasiaticum subsp. ipomoeae' and '*Ca*. P. fabacearum')
*Fabaceae*	*Medicago sativa*	C	'*Ca*. P. fabacearum';New 16SrII species
*Fabaceae*	*Stylosanthes scabra*	C	'*Ca*. P. stylosanthis';'*Ca*. P. australasiaticum' taxon 5;Mixed infection ('*Ca*. P. australasiaticum subsp. ipomoeae' and '*Ca*. P. stylosanthis')
*Fabaceae*	*Vigna radiata*	C	'*Ca*. P. australasiaticum subsp. ipomoeae'
*Fabaceae*	*Vigna unguiculata* ssp. *sesquipedalis*	C	'*Ca*. P. australasiaticum subsp. ipomoeae';'*Ca*. P. fabacearum'
*Lecythidaceae*	*Planchonia careya*	NP	'*Ca*. P. planchoniae'
*Malvaceae*	*Sida* sp.	W	'*Ca*. P. australasiaticum subsp. australasiaticum';'*Ca*. P. australasiaticum' taxon 4
*Malvaceae*	*Waltheria* sp.	W	*Waltheria* Little Leaf phytoplasma
*Myrtaceae*	*Melaleuca* sp.	NP	'*Ca*. P. melaleucae'
*Rosaceae*	*Fragaria ×Ananassa Duch. Cv. Pajaro*	C	'*Ca*. P. australasiaticum' taxon 1
*Solanaceae*	*Capsicum annuum*	C	'*Ca*. P. australasiaticum subsp. australasiaticum';'*Ca*. P. australasiaticum subsp. ipomoeae';'*Ca*. P. australasiaticum' taxon 1;'*Ca*. P. fabacearum'
*Solanaceae*	*Petunia* sp.	O	'*Ca*. P. australasiaticum subsp. australasiaticum'
*Solanaceae*	*Solanum lycopersicum*	C	'*Ca*. P. australasiaticum subsp. australasiaticum';'*Ca*. P. australasiaticum subsp. ipomoeae'; '*Ca*. P. fabacearum'
*Solanaceae*	*Solanum melongena*	C	'*Ca*. P. australasiaticum subsp. australasiaticum';'*Ca*. P. australasiaticum subsp. ipomoeae';'*Ca*. P. australasiaticum' taxon 1
*Solanaceae*	*Solanum nigrum*	W	'*Ca*. P. australasiaticum subsp. ipomoeae'
*Solanaceae*	*Solanum* sp.	W	'*Ca*. P. australasiaticum subsp. australasiaticum';'*Ca*. P. australasiaticum' taxon 1;'*Ca*. P. australasiaticum' taxon 2;Mixed infection ('*Ca*. P. australasiaticum' taxon one and '*Ca*. P. stylosanthis')
*Solanaceae*	*Solanum tuberosum*	C	'*Ca*. P. australasiaticum subsp. australasiaticum'; '*Ca*. P. stylosanthis'
*Vitaceae*	*Vitis viniferacv*. Chardonnay*/Riesling*	C	'*Ca*. P. australiense' (16SrXII)(blastn);16SrII Alfalfa phytoplasma (Sudan) (blastn)
**New phytoplasma host records for Australia from this study**			
*Apiaceae*	*Petroselinum crispum*	C	'*Ca*. P. australasiaticum subsp. australasiaticum'
*Asteraceae*	*Bidens pilosa*	W	'*Ca*. P. australasiaticum subsp. ipomoeae'
*Asteraceae*†	*Chromolaena odorata*	W	Mixed infection ('*Ca*. P. australasiaticum subsp. ipomoeae' and '*Ca*. P. fabacearum')
*Asteraceae*	*Gynura crepioides* (Okinawa Spinach)	C	'*Ca*. P. australasiaticum' taxon 1
*Asteraceae*	*Osteospermum* sp.	O	'*Ca*. P. australasiaticum subsp. australasiaticum'
*Asteraceae*	*Peripleura diffusa*	NP	'*Ca*. P. australasiaticum subsp. ipomoeae'
*Asteraceae*	*Praxelis clematidea*	W	'*Ca*. P. australasiaticum subsp. ipomoeae';Mixed infection ('*Ca*. P. australasiaticum subsp. australasiaticum' and '*Ca*. P. fabacearum')
*Convolvulaceae*	*Convolvulus clementii*	C	'*Ca*. P. australasiaticum subsp. australasiaticum'
*Cucurbitaceae*	*Citrullus lanatus*	C	'*Ca*. P. australasiaticum subsp. ipomoeae'
*Cucurbitaceae*	*Cucumis sativus*	C	'*Ca*. P. australasiaticum subsp. ipomoeae'
*Cucurbitaceae*	*Luffa acutangula*	C	'*Ca*. P. australasiaticum subsp. ipomoeae'
*Cucurbitaceae*	*Luffa* sp.	C	'*Ca*. P. australasiaticum subsp. ipomoeae'
*Cucurbitaceae*	*Momordica charantia*	C	'*Ca*. P. australasiaticum subsp. ipomoeae';ViLL
*Cucurbitaceae*	*Trichosanthes cucumerina*	C	'*Ca*. P. australasiaticum' taxon 3
*Fabaceae*	*Bituminaria bituminosa*	C	'*Ca*. P. fabacearum'
*Fabaceae*	*Crotalaria juncea*	W	'*Ca*. P. australasiaticum subsp. ipomoeae'
*Geraniaceae*	*Geranium* sp.	O	'*Ca*. P. australasiaticum subsp. australasiaticum'
*Geraniaceae*	*Pelargonium* sp.	O	'*Ca*. P. australasiaticum subsp. ipomoeae'
*Asteraceae*	*Echinacea purpurea*††	O	'*Ca*. P. australasiaticum subsp. australasiaticum'
*Cucurbitaceae*	*Cucurbita pepo var. giromontiina* (Zucchini)††	C	'*Ca*. P. australasiaticum subsp. ipomoeae'
*Goodeniaceae*	*Goodenia scaevolina*††	NP	Mixed infection ('*Ca*. P. australasiaticum subsp. australasiaticum' and '*Ca*. P. stylosanthis')
*Malvaceae*	*Gomphocarpus fruticosus*††	wW	'*Ca*. P. australasiaticum subsp. australasiaticum'
*Pedaliaceae*	*Phyllanthus fuernrohrii*††	NP	'*Ca*. P. australasiaticum subsp. ipomoeae'

*Indicates a sample for which the initial host family was unknown based on morphology but was subsequently identified to be a species within the *Asteraceae* using plant DNA barcodes.

†Indicates a sample where the host was initially identified as a *Lamiaceae* sp. but was subsequently identified as an *Asteraceae* species.

‡Indicates a sample for which the host was initially identified as a *Fabaceae* species but was subsequently identified to be a species within the genus *Crotalaria*.

§Indicates a sample where the host was initially identified to be a *Lamiaceae* species but for which the plant barcodes identified the host as a *Malvaceae* species (genus: *Sida*).

¶Indicates a sample for which the host species was unknown but subsequently identified as a species within the *Solanaceae* family.

**Indicates samples where an insect host was identified to the genus *Orosius* but which was subsequently identified as *Orosius argentatus* based on analysis of the COI gene sequence.

††Indicates a new host species for phytoplasmas in Australia but not a new host genus record.

Of the 24 phytoplasma-positive weed samples, 16 were unidentified to the genus- or species-level based on morphology or DNA barcode analysis (Tables3  4). These 16 samples included species in the plant families *Asteraceae* (*Praxelis clematidea*, *n*=2; *Bidens pilosa*, *n*=1), *Convolvulaceae* (*Bonamia pannosa*, *n*=2; *Ipomoeae* sp., *n*=1), *Fabaceae* (*Crotalaria juncea*, *n*=2; *Crotalaria* sp., *n*=1), *Malvaceae* (*Gomphocarpus fruticosus*, *n*=3; *Waltheria* sp., *n*=1), and the *Solanaceae* (*Solanum nigrum*, *n*=2; *Solanum* sp., *n*=1). Ornamental hosts included species in the families *Apocynaceae* (*Catharanthus roseus*, *n*=2), *Asteraceae* (*Echinaceae purpurea*, *n*=1; *Osteospermum* sp., *n*=1), *Geraniaceae* (*Geranium* sp., *n*=1; *Pelargonium* sp., *n*=1), and *Solanaceae* (*Petunia* sp., *n*=1) (Table 4).

Native plants included those in the *Asteraceae* (*Peripleura diffusa*, *n*=1), *Goodeniaceae* (*Goodenia scaevolina*, *n*=1), *Lecythidaceae* (*Planchonia careya*, *n*=1), *Myrtaceae* (*Melaleuca* sp., *n*=2), and *Pedaliaceae* (*Phyllanthus fuernrohrii*, *n*=1) families (Table 4).

All three insects in this study from which phytoplasmas were detected were species of the *Cicadellidae* family, with one identified as *Orosius orientalis* (BAWM-232), and the other two identified as *Orosius argentatus* (BAWM-342B and BAWM-343A) (Table 4). ‘*Ca*. Phytoplasma australasiaticum subsp. australasiaticum’, ‘*Ca*. Phytoplasma australasiaticum subsp. ipomoeae’, and ‘*Ca*. Phytoplasma stylosanthis’ were detected in *Orosius* species (family: Cicadellidae) analysed in this study (Tables1^3^ and S1). *Orosius* species, including *Orosius argentatus*, have been identified as confirmed or putative vectors of diseases thought to be associated with ‘*Ca*. Phytoplasma australasiaticum subsp. australasiaticum’ and ‘*Ca*. Phytoplasma stylosanthis’, such as tomato big bud, tobacco little leaf, and legume little leaf diseases [15,27, 29]. The detection of ‘*Ca*. Phytoplasma australasiaticum subsp. australasiaticum’ and ‘*Ca*. Phytoplasma stylosanthis’ in *Orosius argentatus* in this study (Tables 1 and S1) is therefore consistent with the results of previous collection efforts in Australia [15] and it may be that this leafhopper species is a vector of several phytoplasma taxa. However, the detection of phytoplasma strains from the total nucleic extracts from insect whole bodies or subsampled body sections, as done in this study, does not provide definitive evidence of vector competence and further transmission trials are required. A comprehensive list of insect species that serve as competent vectors of phytoplasma diseases of vegetable crops in Australia remains to be determined.

#### Summary of new phytoplasma host records for Australia

Of the 56 unique species sampled in this study, 23 were recorded as new phytoplasma hosts for Australia (Tables 4 and S1). Eleven different plant genera were determined to be new phytoplasma hosts for Australia (Tables 4 and S1), and included the species *Bidens pilosa*, *Citrullus lanatus*, *Convolvulus clementii*, *Geranium* sp., *Pelargonium* sp., *Gynura crepioides* (Okinawa spinach), *Momordica charantia*, *Osteospermum* sp., *Peripleura diffusa*, *Praxelis clematidea*, and *Trichosanthes cucumerina*. Plant species identified as new phytoplasma host reports but for which the genus was already known to be a host in Australia (*n*=5), included *Crotalaria juncea* (previous detection reported as *Crotalaria goreensis* or *Crotalaria novae-hollandiae* [12]; or as *Crotalaria* sp. [8,12]), *Echinacea purpurea* (previous detection in *Echinacea pallida* [69]), *Gomphocarpus fruticosus* (previous detection in *Gomphocarpus physocarpus* [70]), *Goodenia scaevolina* (previous detection reported as *Goodenia* sp. [13]), and *Phyllanthus fuernrohrii* (previous detection in *Phyllanthus amarus* [71]) (Tables 4 and S1).

#### Group 16SrII phytoplasma taxa

Species classified within the 16SrII group were detected in a broad range of host species and were the most frequently detected from the geographic regions and vegetable crops investigated in this study (Figs1  2) (Tables 4 and S1). A total of 12 16SrII taxa were identified in the samples analysed and included: ‘*Ca*. Phytoplasma australasiaticum subsp. australasiaticum’, ‘*Ca*. Phytoplasma australasiaticum subsp. ipomoeae’, ‘*Ca*. Phytoplasma australasiaticum’ taxon 1 to taxon 5, ‘*Ca*. Phytoplasma bonamiae’, ‘*Ca*. Phytoplasma fabacearum’, ‘*Ca*. Phytoplasma planchoniae’, the WaLL phytoplasma, and the potentially new 16SrII species (Fig. 1).

##### ‘*Ca.* Phytoplasma australasiaticum subsp. australasiaticum’ and ‘*Ca.* Phytoplasma ustralasiaticum subsp. pomoeae’ (16SrII)

**Fig. 3.**
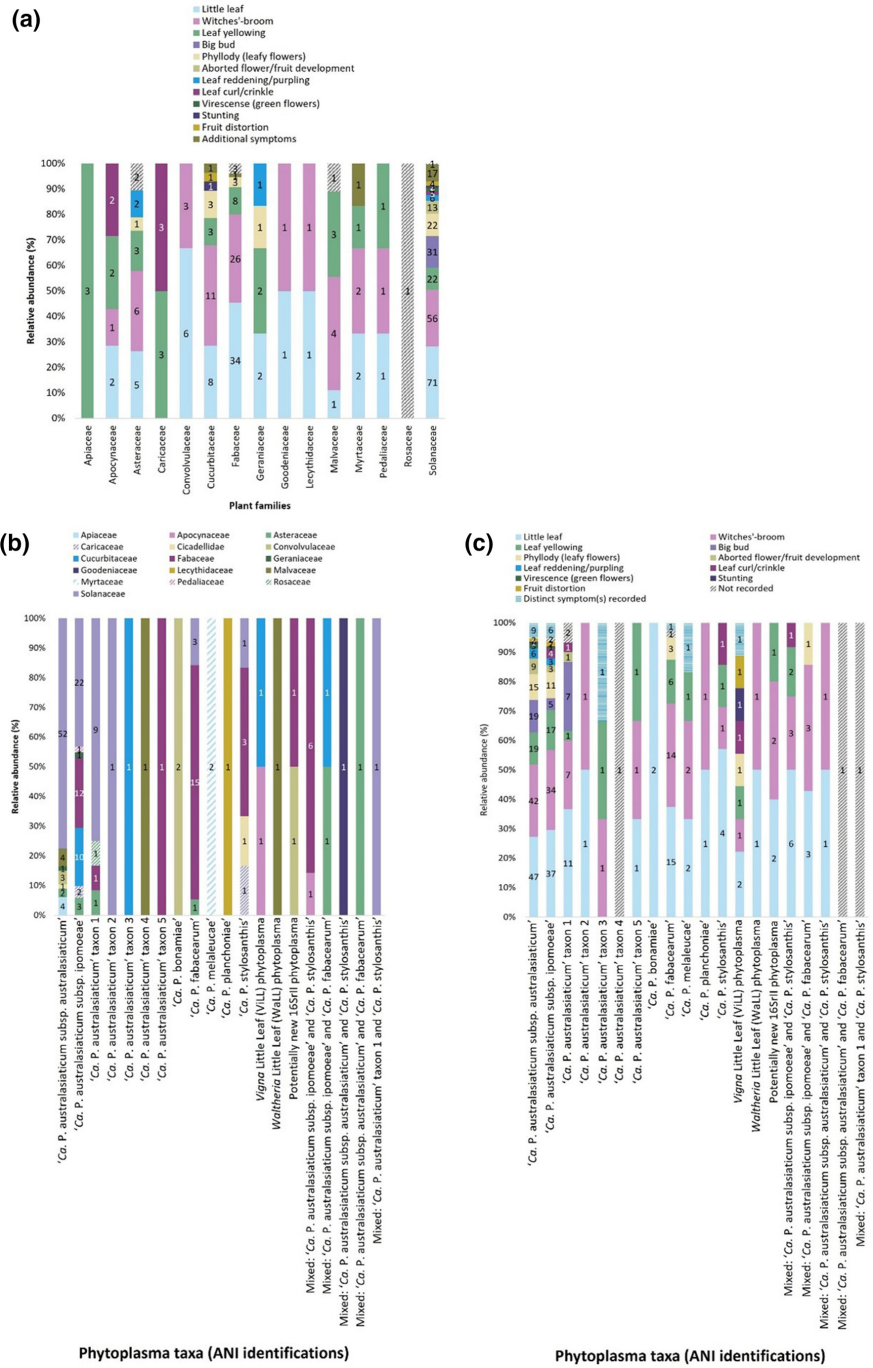
Bar graphs indicating the relative abundances of (**a**) symptom types recorded for each plant host family analysed in this study (*n*=176 samples); (**b**) the ANI-identified phytoplasma taxa per plant or insect host family analysed in this study (*n*=178 samples); and (**c**) the symptom types recorded for each ANI-identified phytoplasma taxon analysed in this study (*n*=176 samples). Numbers in the bar graphs indicate the total number of samples. Colour legends are shown above each graph.

‘*Ca*. Phytoplasma australasiaticum subsp. australasiaticum’ and ‘*Ca*. Phytoplasma australasiaticum subsp. ipomoeae’ were detected in all five states/territories investigated in this study (Table 1; Fig. 2). These two subspecies were also the most abundant taxa sampled in each state (Fig. 2). ‘*Ca*. Phytoplasma australasiaticum subsp. australasiaticum’, was detected in seven host families (Fig. 3a, b). ‘*Ca*. Phytoplasma australasiaticum subsp. australasiaticum’ was identified in single infections in nine of the 34 new phytoplasma host species or genera identified in this study. New phytoplasma host records for ‘*Ca*. Phytoplasma australasiaticum subsp. australasiaticum' included the new phytoplasma hosts for Australia in the plant families *Apiaceae* (*Petroselinum crispum*), *Asteraceae* (*Echinaceae purpurea*, *Osteospermum* sp.), *Convolvulaceae* (*Convolvulus clementii*), *Geraniaceae* (*Geranium* sp.), and *Malvaceae* (*Gomphocarpus fruticosus*) (Table 4). This phytoplasma was associated with 11 symptom types, including little leaf, witches’-broom, yellowing, big bud, phyllody, and stunting (Fig. 3c and Table S1).

‘*Ca*. Phytoplasma australasiaticum subsp. ipomoeae’ was detected from samples classified into seven host families (Fig. 3b) and was associated with symptoms of little leaves, witches’-broom, leaf discolouration (yellowing, purpling/reddening), big bud, phyllody, aborted flower/fruit development, fruit distortions and leaf curl/crinkle (Fig. 3c and Table S1). ‘*Ca*. Phytoplasma australasiaticum subsp. ipomoeae’ was identified in single infections in 12 of the 34 new phytoplasma host species or genera identified in Australia (Table 4), including those in the *Asteraceae* (*Peripleura diffusa*, *Praxelis clematidea*), *Cucurbitaceae* (*Citrullus lanatus; Momordica charantia*, *Luffa acutangular*, a *Luffa* sp.), *Fabaceae* (*Crotalaria juncea*), *Geraniaceae* (*Pelargonium* sp.), and *Pedaliaceae* (*Phyllanthus fuernrohrii*).

The observation that ‘*Ca*. Phytoplasma australasiaticum subsp. australasiaticum’ and ‘*Ca*. Phytoplasma australasiaticum subsp. ipomoeae’ were the most frequently detected, the most widespread geographically, and detected from the most host families and species in this study is not surprising (Figs1^3^). These two species have historically been detected from a broad range of hosts in Australia, including many crop species [27]. ‘*Ca*. Phytoplasma australasiaticum subsp. australasiaticum’ and ‘*Ca*. Phytoplasma australasiaticum subsp. ipomoeae’ are major threats to crop production in Australia as they are commonly associated with crop species in many vegetable growing areas around the country, and disease incidences can be high [72]. Additionally, the symptoms they are associated with directly affect crop yield (e.g. phyllody). Further research is required to determine whether these two subspecies have distinct host ranges, symptomologies, or vector species for a better understanding of their biology and how to mitigate outbreaks of diseases associated with them.

##### ‘*Ca*. Phytoplasma australasiaticum’ taxon 1 (16SrII)

‘*Ca*. Phytoplasma australasiaticum’ taxon 1 was detected in the NT (*n*=9), QLD (*n*=2), and VIC (*n*=1) (Fig. 2). This taxon was detected for hosts in the families *Solanaceae* (*Solanum melanogena, n*=7 samples; *Solanum* sp. weed, *n*=1 sample), *Asteraceae* (*Gynura crepioides*, *n*=1 sample), *Fabaceae* (*Glycine max*, *n*=1 sample), and *Rosaceae* (*Fragaria*×*Ananassa* Duch. Cv. Pajaro, *n*=1 sample) (Fig. 3b). The detection of ‘*Ca*. Phytoplasma australasiaticum’ taxon 1 in *Gynura crepioides* (family: *Asteraceae*) represents a new phytoplasma host record for Australia (Table S1). The symptoms of the *Fragaria*×*Ananassa* Duch. Cv. Pajaro and *Solanum* sp. hosts were present upon sampling but were not recorded (Fig. 3c and Table S1). Six symptom types were recorded for the 10 remaining hosts, including little leaf (*n*=10), big bud (*n*=7, all *Solanum melanogena* samples), witches’-broom (*n*=5), as well as leaf yellowing, aborted flower/fruit development and leaf curl/crinkle (*n*=1 each, *Capsicum annuum*).

This taxon may present a moderate threat to crop production in Australia, with the potential to affect Solanaceae hosts in particular. This is due to this taxon being detected from several crop hosts displaying symptoms that directly affect crop yield but also due to the large geographic range of the detections made in this study (from the NT, QLD, and VIC). Additionally, the detection of ‘*Ca*. Phytoplasma australasiaticum’ taxon 1 was likely missed in previous RFLP-based analyses done to assess taxon diversity in Australia and may, therefore, have a broader host and geographic range than what is reported in this study. Further research is required to investigate the prevalence and vector(s) of this taxon.

##### ‘*Ca.* Phytoplasma australasiaticum’ taxa 2 to 5 (16SrII)

‘*Ca*. Phytoplasma australasiaticum’ taxon 2 (*n*=1, a Solanaceae weed in the genus *Solanum,* BAWM-057), ‘*Ca*. Phytoplasma australasiaticum’ taxon 4 (*n*=1, Malvaceae weed in the genus *Sida*, BAWM-319), and ‘*Ca*. Phytoplasma australasiaticum’ taxon 5 (*n*=1, *Stylosanthes scabra*, Fabaceae, BAWM-324) were all detected in QLD (Figs2  3b and Table S1). No symptoms were recorded for ‘*Ca*. Phytoplasma australasiaticum’ taxon 4, whereas ‘*Ca*. Phytoplasma australasiaticum’ taxon 2 and 5 both displayed witches’-broom symptoms (Fig. 3c and Table S1). Additional symptoms for these hosts included little leaf symptoms associated with the *Solanum* sp. infected with ‘*Ca*. Phytoplasma australasiaticum’ taxon 2, as well as both little leaf and yellowing for the *Stylosanthes scabra* host infected with ‘*Ca*. Phytoplasma australasiaticum’ taxon 5 (Fig. 3c and Table S1). ‘*Ca*. Phytoplasma australasiaticum’ taxon 3 (BAWM-306) was detected in the NT from a *Trichosanthes cucumerina* (Cucurbitaceae) sample (Figs2  3b; Table S1). The phytoplasma was associated with witches’-broom and yellowing, as well as ‘shoe stringing’ of the leaves (Fig. 3c and Table S1).

The ANI results indicated that these taxa were distinct and are likely novel subspecies of ‘*Ca*. Phytoplasma australasiaticum’ (Fig. 1), representing the first time these taxa have been identified in Australia and likely globally. These novel detections are potentially due to these identifications being missed previously due to the high sequence similarity of their 16S rRNA gene to other described 16SrII phytoplasma taxa (>99 % nucleotide sequence similarity; Table S1). Additionally, these novel detections may not have been made in the past as four of these novel phytoplasma taxa were detected from either weed species or from phytoplasma crop host species that are first records of phytoplasma hosts for Australia (Table 4).

The potential threat of these phytoplasma taxa remains to be determined as only a few detections in plant hosts were made in this study and their detections were likely missed in previous low resolution (RFLP-based) analyses (Table S1). Additionally, no vector species have been identified for these taxa. These results highlight the importance of sampling weed species in and around cropping areas, as well as collecting diverse species of symptomatic hosts in an area. These taxa need to be assessed further to determine whether they are truly distinct subspecies, which can be done using further comparative and phylogenomic assessments in future [36,37].

##### ‘*Ca.* Phytoplasma fabacearum’ (16SrII)

‘*Ca*. Phytoplasma fabacearum’ was detected in the NT (*n*=1), QLD (*n*=8), and WA (*n*=7) (Fig. 2). ‘*Ca*. Phytoplasma fabacearum’ was detected in nine distinct plant species and was most frequently detected in *Fabaceae* species (*n*=15 samples, *Fabaceae* species: *Cajanus cajan*, *Cicer arietinum*, *Glycine max*, *Medicago sativa*, *Stylosanthes scabra*, *Bituminaria bituminosa*, and *Vigna unguiculata* ssp. *sesquipedalis*; Fig. 2b and Table S1). ‘*Ca*. Phytoplasma fabacearum’ was also detected in three Solanaceae samples (Solanaceae species: *Capsicum annuum* and *Solanum lycopersicum*), and one Asteraceae sp. sample (Fig. 3b and Table S1). In these hosts, ‘*Ca*. Phytoplasma fabacearum’ was associated with symptoms of little leaf, witches’-broom, yellowing, and phyllody, but also the distinct symptom of clearing of new leaf growth for one *Capsicum annuum* sample (Fig. 3c and Table S1).

These results highlight that *Fabaceae* crops across a broad geographic range in Australia are at a high risk of losses due to infection by ‘*Ca*. Phytoplasma fabacearum’, although some *Asteraceae, Cucurbitaceae*, and *Solanaceae* hosts might also be at risk. This is also supported by reports of high incidence of phytoplasma diseases in Australia likely attributed, in part, to ‘*Ca*. Phytoplasma fabacearum’ [72].

##### Historic samples: ‘*Ca.* Phytoplasma bonamiae’ and the *Waltheria* Little leaf phytoplasma (16SrII)

Strains of ‘*Ca*. Phytoplasma bonamiae’ (*n*=2) identified from *Bonnamia pannosa* and the WaLL phytoplasma (*n*=1) identified from a *Waltheria* sp. were only identified from the historically collected samples analysed in this study from QLD and the NT, respectively (Figs2  3b, c; Table S1). No new host species or geographic range expansions are, therefore, reported for these taxa. ‘*Ca*. Phytoplasma bonamiae’ was associated with little leaf symptoms in both samples, while the WaLL phytoplasma was associated with both little leaf and witches’-broom symptoms. No insect vector species have been identified for these phytoplasma taxa.

This study provides a full-length sequence of the 16S rRNA gene as well as genomic data for the WaLL phytoplasma for the first time. This sequence data identified this phytoplasma as a member of the 16SrII group (Figs 1 and S1; Table S1), which confirms previous reports based on nucleotide analysis of regions within the 16S rRNA gene [13]. Additionally, 16 Sr rRNA and ANI sequence analysis showed that the ‘*Ca*. Phytoplasma bonamiae’ and WaLL phytoplasmas were close relatives of the ‘*Ca*. Phytoplasma fabacearum’ strains (Figs1  2) [13]. However, since both these phytoplasma taxa are infrequently detected in crop plants, and since ‘*Ca*. Phytoplasma bonamiae’ has only been detected from the Australian native plant *Bonamia pannosa* (based on 14 samples in the NT DITT database and [15,27]), they are both unlikely to pose a major threat to crop production in Australia.

It is likely that the WaLL phytoplasma strain can be described as a novel species as it shares less than 96 % ANI with other phytoplasma species (Fig. 1). Eight WaLL strains are in the NT DITT database and future work can investigate whether these WaLL phytoplasma strains further fulfil the updated guidelines to be described as a novel ‘*Ca*. Phytoplasma’ species should more sequence data be made available for them [31].

##### ‘*Ca.* Phytoplasma planchoniae’ (16SrII)

‘*Ca*. Phytoplasma planchoniae’ was detected from a *Planchonia careya* host sampled in QLD (Figs2  3b) that displayed little leaves and witches’-broom symptoms (Fig. 3c). ‘*Ca*. Phytoplasma planchoniae’ has previously been detected in Australia and has only been associated with native plant *Planchonia careya* in far north QLD [73]. Due to its narrow host range in a non-crop species and its restricted geographic range, ‘*Ca*. Phytoplasma planchoniae’ is unlikely to pose a major threat to crop production in Australia.

##### Potentially new 16SrII species

Strains of the potentially new 16SrII species were detected from an *Ipomoea* sp. (*Convolvulaceae*, sample BAWM-339) and from a *Medicago sativa* sample (*Fabaceae*, sample BAWM-167) from the NT and WA, respectively (Figs.2  3b; Table S1). Both hosts showed symptoms of little leaf and witches’-broom, but the *Medicago sativa* sample BAWM-167 also showed symptoms of yellowing (Fig. 3c and Table S1). While further investigations are required to determine whether these two strains belong to a novel ‘*Candidatus* Phytoplasma’ species within the 16SrII group, support for their delimitation include the observation that (i) they produced ANI and AF values below the within-species threshold (<95 % and <80 %) with already described ‘*Candidatus* Phytoplasma’ species, and (ii) more than one strain of this potential species was identified from distinct hosts from different areas in Australia. It remains to be determined what threat to crop production in Australia this taxon presents as only these two strains were identified in this study with the competent or putative vector species unknown.

### Group 16SrXXXVIII Phytoplasma samples – ‘Ca. Phytoplasma stylosanthis’

‘*Ca*. Phytoplasma stylosanthis’ was detected in four of the five states/territories, including the NT (*n*=1), NSW (*n*=1), QLD (*n*=3), VIC (*n*=1), and WA (*n*=1) (Fig. 2). This phytoplasma was detected as single phytoplasma infection from four host families, namely the *Caricaceae* (*n*=1, *Carica papaya*), *Cicadellidae* (*n*=1, *Orosius argentatus*), *Fabaceae* (*n*=3*, Stylosanthes scabra*), and *Solanaceae* (*n*=1, *Solanum tuberosum*) (Fig. 3b and Table S1). Four of the five ‘*Ca*. Phytoplasma stylosanthis’-infected plant hosts displayed little leaf symptoms, with the one sample, that did not show little leaf symptoms, showing yellowing and leaf curl/crinkle symptoms instead (BAWM-249, *Carica papaya*) (Fig. 3c). The *Solanum tuberosum* sample had the additional symptom of witches’-broom (Fig. 3c).

Since ‘*Ca*. Phytoplasma stylosanthis’ had previously only been reported in NT, QLD, and NSW [13,18, 74], the *Solanum tuberosum* sample represents both a host and geographic range expansion for this phytoplasma (Table S1) as described previously (sample VPRI 43683 [24]). Since this phytoplasma has been identified from a broad range of crop species across a large geographic area, ‘*Ca*. Phytoplasma stylosanthis’ has the potential to be associated with reductions of economically important crops such as *Carica papaya* [15,27].

### Group 16SrXXV Phytoplasma samples – ‘Ca. Phytoplasma melaleucae’

‘*Ca*. Phytoplasma melaleucae’ was detected in QLD (*n*=1; BAWM-155) and WA (*n*=1; BAWM-354) (Fig. 2) and were only detected as single infections from *Melaleuca* spp. (Myrtaceae) in Australian regions above the Tropic of Capricorn (Tables 1 and S1). This is the first report of ‘*Ca*. Phytoplasma melaleucae’ for WA and the furthest west occurrence of this phytoplasma. Prior to the present study, this phytoplasma was only reported from far north QLD and the Western Province of Papua New Guinea in *Melaleuca* spp., with one case reported for *Synsepalum dulcificum* (Sapotaceae) [16,37]. The two samples analysed in this study displayed little leaf and witches’-broom symptoms (Fig. 3c and Table S1), which is consistent with previous detections [16]. At present, this phytoplasma is unlikely to pose a major threat, if any, to vegetable crop production in Australia due to its restricted host range to non-crop hosts.

### Phytoplasma 16Sr group unassigned – vigna little leaf phytoplasma

The ViLL phytoplasma was detected in the NT and WA (*n*=1 per state/territory; Fig. 2). Sample BAWM-245 represents a host and geographic range expansion for the ViLL phytoplasma, being detected for the first time in WA and in a *Catharanthus roseus* sample (*Apocynaceae*; Table S1; [27]). A second host expansion for this phytoplasma and new phytoplasma host for Australia was *Momordica charantia* (*Cucurbitaceae*), detected in the NT (sample BAWM-336) where there was a high incidence of disease (70–80 % of crop affected, in-field observation by S. Bond). Prior to this study, this phytoplasma taxon was only reported in Australia from within or near Katherine and Darwin in the NT [13,15, 74]. Both samples showed little leaf symptoms, however the *Catharanthus roseus* sample (BAWM-245) also showed symptoms of leaf yellowing and leaf curl/crinkle, while the *Momordica charantia* sample (BAWM-336) showed additional symptoms of witches’-broom, phyllody, stunting, and fruit distortions (Fig. 3c and Table S1).

ViLL phytoplasmas have been reported from symptomatic *Tridax procumbents* (family: *Asteraceae*), *Stylosanthes scabra* (family: *Fabaceae*), and *Vigna lanceolata* (family: *Fabaceae*) [13,15, 74], with *Austroagallia torrida* (Evans) (family: *Cicadellidae*) and a *Batracomorphus* species (family: *Cicadellidae*) identified as possible vectors [15]. Although this phytoplasma taxon has been infrequently detected in Australia, the high incidence of disease observed for the crop *Momordica charantia* in the NT (in-field observation by S. Bond) and the broad host range suggests that this phytoplasma could be a threat to crop production in Australia, particularly in regions above the Tropic of Capricorn.

### Group 16SrXII phytoplasma (16S rRNA PCR identification only)

A single ‘*Ca*. Phytoplasma australiense’ (16SrXII) detection was made in this study from a *Vitis vinifera cv*. Chardonnay sample (BAWM-189) collected in VIC (Table S1). This sample did not produce sufficient phytoplasma data to be used in the genome-based analyses despite the application of several approaches aimed at increasing the amount of phytoplasma DNA sequence data obtained from the background ‘contaminating’ DNA. These approaches included sampling different tissue types (e.g. whole leaves and petioles, vs petioles and leaf veins only), the application of the iodixanol-based phytoplasma enrichment method, as well as Illumina sequencing of these samples at a high depth (>22 Gb of data; Table S1). Although only one detection of this phytoplasma species was made in this study, this phytoplasma has previously been detected in a broad range of high-value hosts in Australia, including: *Carica papaya*, *Cucurbita maxima*, *Fragaria*×*Ananassa* Duch. Cv. Pajaro, and *Vitis vinifera* [12,13, 17, 75,77] suggesting that this phytoplasma has the potential to impact crop production in Australia. While attempts have been made to identify the vector species of ‘*Ca*. Phytoplasma australiense’ in Australia [78], they remain to be determined.

### Mixed phytoplasma infections

Mixed infections were identified from a total of 12 plant samples, which were collected in the NT (*n*=1), QLD (*n*=7), and WA (*n*=4) (Fig. 2). Mixed infections of ‘*Ca*. Phytoplasma australasiaticum subsp. ipomoeae’ and ‘*Ca*. Phytoplasma stylosanthis’ were recorded for five samples in QLD (all *Stylosanthes scabra* hosts collected in Mareeba and Atherton in the years 1998 and 2018; Tables 1 and S1; Fig. 2). One mixed infection of ‘*Ca*. Phytoplasma australasiaticum subsp. ipomoeae’ and ‘*Ca*. Phytoplasma stylosanthis’ was recorded for WA in *Catharanthus roseus*, representing both a host and geographic range expansion for ‘*Ca*. Phytoplasma stylosanthis’. Mixed infections involving ‘*Ca*. Phytoplasma fabacearum’ and ‘*Ca*. Phytoplasma australasiaticum subsp. ipomoeae’ were also reported from QLD (*n*=1, *Chromolaena odorata*) and WA [*n*=2, *Cucurbita* sp. (Pumpkin) and *Glycine max*] (Tables 1 and S1; Fig. 2).

Three different mixed infection types were recorded for single plant samples collected either in the NT, QLD, or WA (Fig. 2 and Table S1). One of these included a single *Solanum* sp. sample with a mixed infection involving ‘*Ca*. Phytoplasma australasiaticum’ taxon 1 and ‘*Ca*. Phytoplasma stylosanthis’ collected in the NT (Fig. 2), which represents a new host species for ‘*Ca*. Phytoplasma stylosanthis’ (Fig. 1; Tables 3 and S1). The mixed infection involving ‘*Ca*. Phytoplasma fabacearum’ and ‘*Ca*. Phytoplasma australasiaticum subsp. australasiaticum’ was only detected in a single *Praxelis clematidea* sample collected in QLD (Fig. 2). The mixed infection involving ‘*Ca*. Phytoplasma australasiaticum subsp. australasiaticum’ and ‘*Ca*. Phytoplasma stylosanthis’ was detected in a single *Goodenia scaevolina* sample collected in WA (Fig. 2). *Goodenia scaevolina* represents a new phytoplasma host species record for Australia (Tables 4 and S1).

Although symptoms were present upon sampling, they were not recorded for the sample with the mixed infection of ‘*Ca*. Phytoplasma australasiaticum’ taxon 1 and ‘*Ca*. Phytoplasma stylosanthis’, nor for the mixed infection involving ‘*Ca*. Phytoplasma australasiaticum ipomoeae’ and ‘*Ca*. Phytoplasma fabacearum’ (Fig. 3c; Table S1). The remaining 10 mixed infection samples displayed little leaf symptoms (*n*=10), with witches’-broom symptoms as the next most prevalent symptom (*n*=7), followed by yellowing (*n*=2), phyllody (*n*=1) and leaf crinkle/curl (*n*=1).

Apart from the 16SrII phytoplasmas, ‘*Ca*. Phytoplasma stylosanthis’ was found most frequently in mixed infections with another phytoplasma. Similar instances of mixed infections involving 16SrII phytoplasmas and ‘*Ca*. Phytoplasma stylosanthis’ have been reported previously from both plant and insect hosts [14,15, 74], including mixed infections of ‘*Ca*. Phytoplasma australasiaticum subsp. ipomoeae’ and ‘*Ca*. Phytoplasma stylosanthis’ [74]. These results demonstrate the large geographic and host range of ‘*Ca*. Phytoplasma stylosanthis’ in Australia and its overlapping geographic range with other phytoplasma taxa in the 16SrII group.

### Summary of associations of phytoplasma and host species on symptoms presented in plants

Most of the plant samples displayed little leaf (*n*=138), proliferation/witches’-broom (*n*=120), and/or yellowing symptoms (*n*=57), followed by phyllody (*n*=37), big bud (*n*=31), aborted flower or fruit development (*n*=13), leaf curl or crinkle (*n*=13), leaf reddening or purpling (*n*=9), fruit distortions (*n*=6), virescence (*n*=4), and stunting (*n*=2) (Fig. 3a; Table S1). Additionally, little leaf, witches’-broom, and yellowing symptoms were observed within 13, 12, and 10 of the 14 plant families, respectively, for which symptoms were recorded (Fig. 3a). The most symptom types (*n*=12) were recorded for the plants classified within the *Solanaceae*, followed by those in the *Asteraceae* and *Fabaceae* plant families (*n*=5 each). No symptoms were provided for 12 samples in this study (Fig. 3a; Table S1).

Distinct symptoms were observed and noted for 19 plant hosts (Fig. 3a; Table S1), including ‘shoe stringing’ of the leaves (*Cucurbitaceae* and *Solanaceae* samples BAWM-306 and BAWM-332, respectively), heart-shaped leaves (*Capsicum annuum* samples BAWM-211a-F3 and o7C), and gigantism of the calyx (*Solanum melongena* sample BAWM-003). Nine *Solanum lycopersicum* samples displayed distinct symptoms, including adventitious roots (*n*=5), wilt (*n*=3), and leaf tip necrosis (*n*=1). An interesting observation was the absence of phyllody or virescence for the ViLL phytoplasma-infected *Catharanthus roseus* sample BAWM-245.

No additional symptoms were observed for the mixed infection compared to samples where only a single phytoplasma taxon was observed (Fig. 3c; Table S1). While these associations need to be investigated more thoroughly as only a few host species overlapped between the single and mixed infection samples in this study, these observations have been reported previously [74]. The association of all plant symptoms with phytoplasma infection is, however, difficult to disentangle as phytoplasmas remain to be cultured and, as such, Koch’s postulates cannot be fulfilled. Abiotic factors, herbicide treatments, insect damage, the presence of other microbes, viruses, or a combination of these factors could also contribute to the symptoms that are presented by the plant hosts [79,80].

## Conclusions

In this study, phytoplasma-infected crop and non-crop hosts from historic collections and contemporary collections (2015 to 2022) from vegetable growing regions around Australia were metagenomically sequenced to identify the crop-infecting phytoplasma taxa and potential alternative hosts. A total of 15 distinct phytoplasma taxa were identified from the metagenomic data obtained for these hosts (Figs1  2). ‘*Ca*. Phytoplasma australasiaticum’ subspecies and ‘*Ca*. Phytoplasma stylosanthis’ were two of the most frequently detected taxa identified, and from the broadest range of hosts and locations sampled across Australia (Fig. 2). Additionally, six previously undescribed phytoplasma taxa were identified from the samples analysed in this study, namely: ‘*Ca*. Phytoplasma australasiaticum’ taxon 1 to 5, and a potentially new 16SrII phytoplasma species. A few phytoplasma taxa were infrequently detected in this study, with some only associated with diseases in non-crop plants and, therefore, likely pose a low threat to crop production in Australia (e.g. ‘*Ca*. Phytoplasma melaleucae’). Five different phytoplasma mixed infections were also identified (Figs1  2). An updated list of phytoplasma 16 Sr groups, species, subspecies, and unclassified taxa present in Australian vegetable growing areas, as well as the prevalence and combinations of mixed phytoplasma infections was therefore provided by this study. A list of symptoms per host (Fig. 3a) and per phytoplasma taxon (Fig. 3c) are also provided and, with the previous literature, will aid in-field detections of phytoplasma associated disease in crop and non-crop plant hosts (Table S1).

PCR of the 16S rRNA gene using universal nested phytoplasma primers combined with direct Sanger sequencing was sufficient as a triage tool to screen and provide a preliminary identification of the phytoplasma taxon present in every sample analysed in this study. However, it lacked the taxonomic resolution afforded by the ANI analysis of draft metagenomic-assembled phytoplasma genomes (Fig. S1), emphasizing results from other studies [36]. Additionally, the PCR-based approach often failed to accurately identify mixed infections (Table S1), which has been reported previously for Sanger sequencing of the PCR amplicon obtained directly from a sample [74]. The metagenomic-based approach employed in this study based on whole-genome ANI, however, was able to resolve strains to the subspecies-level and could identify the presence of a mixed phytoplasma infection in a single sample. An additional benefit of using the metagenomic approach during phytoplasma collection was that it allowed for host taxa to be identified through the use of genetic barcodes present in the metagenomic dataset when they were unable to be resolved to the family-, genus-, or species-level based on morphology (Table 3). Together, these results provided more informative data with a more precise assessment of the prevalence and host range of phytoplasmas in vegetable growing regions in Australia compared to previous studies, which could only use RFLP or sequence analysis of the 16S rRNA gene [12,13, 15, 74]. The results presented in this study highlight the benefits of combining metadata (host, location, date, etc.) and metagenomic sequencing for phytoplasma diversity assessments and to understand their epidemiology.

Sufficient phytoplasma genomic data was obtained for 178 (12 mixed infections) of the 195 symptomatic samples for genome based sequence analyses and to be submitted to public sequence repositories (excluding mixed infection samples). The dataset presented here is the largest contribution of phytoplasma genome sequences from a single study to date, increasing the number of publicly available sequences from 47 [81] to a total of 213 (when excluding samples with mixed phytoplasma infections). The incomplete and draft phytoplasma genomes sequenced in this study have significantly increased the taxon sampling of subclade II, which is one of three subclades described in [82] (Fig. S1). The work presented here was possible due to the ever-decreasing cost of HTS and the increased volume of sequence data generated. The phytoplasma genome data obtained in this study can be used in future research to improve phytoplasma taxonomy and diagnostics, and will assist in genomic epidemiology analyses. The reliable genome sequence assemblies will also serve as a resource from which genes involved in symptomology and host/vector interactions can be investigated when combined with the appropriate metadata, as well as comparative and functional analyses. Together, this genome resource will contribute significantly to the knowledge of phytoplasma biology, ecology, and can be used to inform management practises to help mitigate or prevent losses associated with major phytoplasma outbreaks in Australia.

## supplementary material

10.1099/mgen.0.001213 Uncited Supplementary Material 1.

## Abbreviations

ACT: Australian Capital Territory
AF: alignment fraction
ANI: average nucleotide identity
HTS: high throughput sequencing
*mat*K: *maturase* K
MLSA: multilocus sequence analyses
MLST: multilocus sequence typing
NSW: New South Wales
NT: Northern Territory
NT DITT: Northern Territory Department of Industry, Tourism and Trade
QLD: Queensland
*rbc*L: *ribulose-bisphosphate carboxylase*
RFLP: restriction fragment length polymorphism
SA: South Australia
TAS: Tasmania
TBB: Tomato Big Bud
UDI: Unique Dual Index
VIC: Victoria
ViLL: Vigna Little Leaf
WA: Western Australia
WaLL: Waltheria Little Leaf
